# Update and Practical Recommendations for the Use of Medical Treatment of Cushing Syndrome

**DOI:** 10.1210/endrev/bnaf042

**Published:** 2026-01-05

**Authors:** Marta Araujo-Castro, Cristina Lamas, Elisabeth Nowak, John Newell-Price, Martin Reincke, Frederic Castinetti

**Affiliations:** Endocrinology & Nutrition Department, Ramón y Cajal Hospital, 28034 Madrid, Spain; Instituto de Investigación Biomédica Ramón y Cajal (IRYCIS), 28034 Madrid, Spain; Endocrinology & Nutrition Department, Complejo Hospitalario Universitario de Albacete, 02006 Albacete, Spain; Department of Medicine IV, LMU University Hospital, LMU Munich, 81377 Munich, Germany; School of Medicine and Population Health, University of Sheffield, Sheffield S10 T2N, UK; Department of Medicine IV, LMU University Hospital, LMU Munich, 81377 Munich, Germany; School of Medicine and Population Health, University of Sheffield, Sheffield S10 T2N, UK; Aix Marseille Univ, INSERM U1251, Marseille Medical Genetics, 13005 Marseille, France; Assistance Publique Hôpitaux de Marseille, Hopital La Conception, 13005 Marseille, France

**Keywords:** Cushing syndrome, ketoconazole, osilodrostat, levoketoconazole, metyrapone

## Abstract

Medical treatment of hypercortisolism may be necessary for a high proportion of patients with Cushing syndrome (CS), including those who are not candidates for curative surgery. It may also be used in the presurgical period when hypercortisolism is severe, as long-term treatment following surgical failure or recurrence after surgery, or while waiting for the effects of pituitary radiation in Cushing disease. Currently available medical treatments include adrenal steroidogenesis inhibitors that block cortisol secretion (ketoconazole, levoketoconazole, metyrapone, osilodrostat, mitotane, and etomidate), drugs that modulate pituitary ACTH secretion (pasireotide and cabergoline), and drugs that block peripheral glucocorticoid receptors (mifepristone). In addition, there are other medical treatments in development that target pituitary signaling pathways, ACTH or its adrenal receptor, or the conversion of cortisol from cortisone by 11ßHSD1. Steroidogenesis inhibitors can be administered using either a titration or a block-and-replace approach. Titration requires adjusting the daily drug dose with the aim of normalizing circulating cortisol levels, whereas the block-and-replace strategy uses higher drug doses to fully suppress endogenous cortisol production, followed by glucocorticoid supplementation. In this review, we summarize the main indications for medical treatment in CS, the mechanism of drug action, efficacy, recommended doses, and safety of the currently available drugs, as well as potential future treatments. We also discuss titration and block-and-replace approaches for control of hypercortisolism and provide recommendations for the use and monitoring of medical treatment in CS, including patients with endogenous hypercortisolism in special situations such as pregnancy, cyclic CS, and mild autonomous cortisol secretion.

## Essential Points

- Medical treatments for hypercortisolism may be administered preoperatively in cases of severe hypercortisolism, or when surgery has failed, while waiting for the effects of radiation techniques to take place, or when metastases are present
- The choice of cortisol-lowering medication should be based on an individualized approach that takes into account gender, age, secretion levels, and expected treatment outcomes
- Steroidogenesis inhibitors can be effective quickly as single agents or in combination
- Steroidogenesis inhibitors can be used with a titration or block-and-replace approach
- Patients should be systematically educated about the signs and symptoms of adrenal insufficiency while on cortisol lowering drugs
- Promising future compounds may target the MC2R, glucocorticoid receptor, ACTH, or the conversion of cortisone to cortisol by 11-beta HSD1, as well as pathways involved in the pathogenesis of corticotrophic tumors

Cushing syndrome (CS) is a systemic condition caused by chronic and inappropriate exposure to excess glucocorticoids (1). Endogenous CS is rare, with an estimated global incidence of 1.8 to 4.5 cases per million individuals per year (2). Sixty-five percent of endogenous CS is due to a pituitary corticotroph tumor that secretes dysregulated excess of ACTH, known as Cushing disease (CD). Adrenal CS accounts for 30% of all cases of CS and the other 5% are caused by neoplasms outside the pituitary that secrete ACTH—ectopic CS (ECS) (3).

Surgery is considered the first-line treatment for all causes of CS. For CD, remission rates after primary transsphenoidal surgery are around 80% (95% CI, 77-82) (4). Nonetheless, 20% to 30% of those initially in remission experience recurrence during follow-up (5). In adrenal CS, 100% of the cases caused by unilateral benign adrenal adenoma are cured by complete unilateral adrenalectomy, while surgical remission cannot be achieved in stage IV cortisol-producing adrenocortical carcinomas (ACC) or in most of the patients with overt CS and bilateral adrenal disease when unilateral adrenalectomy is performed. In relation to ACC, it should be noted that about 50% to 60% of the patients have clinical hormone excess, and hypercortisolism or mixed Cushing and virilizing syndromes are the most frequently observed. For these patients, mitotane is effective in controlling hypercortisolism, but its efficacy is delayed by several weeks; thus, other medical therapies are usually needed (6). Similarly, ECS caused by benign tumors may be generally cured with the resection of the ACTH/corticotropin-releasing hormone (CRH)-secreting tumor, whereas targeted medical treatment is needed for metastatic ACTH/CRH-producing tumors (7). Overall, medical treatment may be necessary for a high proportion of patients with CS. This includes scenarios when primary surgery is not possible or inappropriate, when presurgical preparation is needed in cases of severe hypercortisolism, when on a long-term basis medical treatment is required after failure or recurrence after surgery, or while waiting for the effects of pituitary radiation to eventually control hypercortisolism in patients with CD. Accurate and timely diagnoses and treatment are essential prerequisites for reducing glucocorticoid-associated complications and improving the quality of life of patients. The main goals of medical therapy are to normalize cortisol secretion and minimize clinical symptoms and comorbidities, improve quality of life, and decrease mortality. Control of pituitary tumor growth is also an important aim of some medical treatments in patients with CD (8).

In this article, we outline the main indications for medical treatment in the management of CS and provide an updated overview of the available options, including the description of different therapeutic approaches (titration and block-and-replace strategies). In addition, we offer recommendations for the selection of the different medical treatments and suggest how best to monitor their efficacy. Finally, we provide recommendations for the treatment of patients with endogenous hypercortisolism in special situations such as pregnancy, cyclic CS and mild autonomous cortisol secretion (MACS).

## Indications for Medical Treatment in Cushing's Syndrome

### Cushing's Disease

#### Classical therapeutic approach

**Figure 1.**
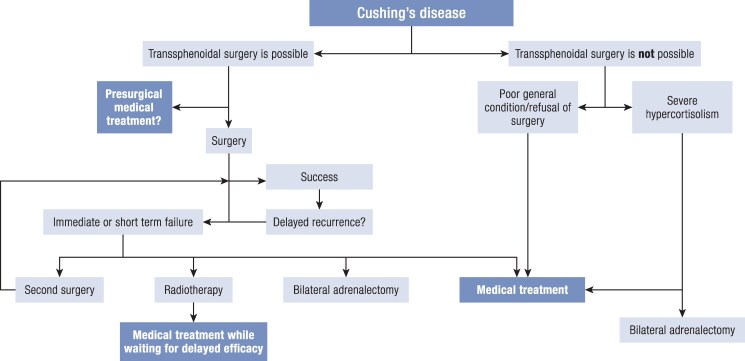
Treatment approach of Cushing disease. Note that medical therapy should be tested before radiotherapy to be sure it is effective and then given until radiotherapy is effective (delayed efficacy).

The first-line treatment for CD is transsphenoidal surgery (9). The detailed diagnostic strategy for ACTH-dependent CS has been presented in recent guidelines and is beyond the scope of this review. In brief, most expert centers consider surgery after confirming the pituitary origin of ACTH secretion even in patients with inconclusive pituitary magnetic resonance imaging (5, 10). In CD, therefore, medical treatment is mainly used for patients who are not in remission after surgery or in the 20% to 30% of patients who experience delayed recurrence after initial surgical remission (11, 12). In such patients, the medical option should be evaluated alongside three other therapeutic options, each of which may provide a permanent remission (Fig. 1 (13)):

1. **A second transsphenoidal surgery,** which can achieve remission in up to 75% to 80% in Pituitary Tumors Centers of Excellence (14, 15), depending largely on the visibility and location of the residual adenomatous tissue and the experience of the neurosurgeon.
2. **Bilateral adrenalectomy**, which leads to almost 100% remission but results in permanent adrenal insufficiency (AI) (16, 17), or
3. **Pituitary radiotherapy** (5), where the time for the effects of radiation to achieve normal cortisol levels can range between 1 and 10 years, necessitating effective medical treatment as bridging therapy (18). Medical treatment is periodically withdrawn to assess the efficacy of radiation.

The definition of recurrence itself is debated and thus deciding when to initiate therapy depends on individual patient characteristics and clinical judgment, especially when recurrent disease is mild. Three different biological markers can be used to define recurrence: a mean of at least two 24-hour urinary free cortisol (UFC) measurements, an average of at least 3 late night salivary cortisol (LNSC) levels, or serum or salivary cortisol following a 1-mg overnight dexamethasone suppression test. Patients who show an increase in at least 2 of these 3 markers are more likely to benefit from medical treatment (1). However, some patients may experience symptoms of recurrence when only 1 of these markers is abnormal. In this situation, the decision to initiate medical treatment should be individualized. As detailed later in this manuscript, determining eucortisolism in patients receiving medical treatment is challenging when all hormonal parameters are compared to elevated levels prior to treatment. It becomes very challenging, therefore, when only one of these parameters is deemed pathological (11).

Finally, in rare cases, patients with pituitary carcinoma may receive medical treatment to control metastatic secretion in a multimodal approach, with temozolomide as the main treatment (19).

#### The role of preoperative medical treatment

In most cases of CD, hypercortisolism is usually mild to moderate, but patients presenting with severe hypercortisolism should be considered with the same approach as discussed in the ECS section. Only a few retrospective studies have analyzed the outcome of patients treated with steroidogenesis inhibitors before surgery (20). The theoretical benefit is to improve comorbidities and reduce the risk of immediate-/short-term complications of surgery. In patients without such comorbidities and who can proceed to surgery without delay, the benefit of a preoperative medical approach is likely to be small or nonexistent. In patients with comorbidities or for whom surgery would be delayed (as happened during the COVID pandemic), medical treatment may be a good option. However, it may take 6 to 12 months to control/normalize metabolic parameters (21), and medical treatment may therefore be needed for the same duration. The benefit of presurgical medical treatment for up to 1 year has never been evaluated, as shown by the ERCUSYN registry, where the median duration of medical treatment was 3 months (for a total of 20% of the 1163 patients in the registry) (22). The most recent expert consensus for the management of CD does not support the systematic use of preoperative medical treatment except in patients in poor general health, or in those for whom there are contraindications for, or who refuse surgery (5).

### Ectopic Cushing Syndrome

In occult ECS, the tumor is not visible on initial imaging. Medical therapy may be given in the first instance, combined with continued imaging every 6 to 12 months, while waiting to see if the tumor becomes visible (eg, by repeated functional positron emission tomography imaging) and surgery can then be considered. In this circumstance, surgery is usually curative, as these tumors are mostly nonmetastatic, well-differentiated neuroendocrine tumors (23).

In ECS with a visible tumor, surgery is usually the first line of treatment (7). However, some patients may present with a very severe hypercortisolism with life-threatening consequences (7). These patients, who present with UFC greater than 10 times the upper limit of normal (ULN), have long been treated with emergency bilateral adrenalectomy to control severe comorbidities such as hypertension, hypokalemia, severe depression, or pulmonary embolism (24). However, in this setting, surgery carries high risk and a mortality of at least 1% to 2% (17). Over the past 5 to 15 years, alternative approaches based on a single highly potent steroidogenesis inhibitor (eg, osilodrostat) or a combination (eg, ketoconazole and metyrapone) have shown very rapid efficacy in controlling cortisol levels and improving comorbidities (or at least allowing control of these comorbidities with high-dose symptomatic treatments) (25-27). The first-line treatment of hypercortisolism for such tumors with high cortisol levels should thus be medical treatment with a rapid dose escalation to allow patient optimization for timely surgery to remove the causative tumor or bilateral adrenalectomy. The medical treatment is frequently given as a block-and-replace approach, as detailed later. In patients with metastases at diagnosis, or during the course of the disease, medical treatment can be provided together with multimodal therapies including debulking surgery, ablation techniques, chemotherapy, and peptide receptor radiotherapy. Bilateral adrenalectomy still plays an important role in such cases (28).

### Adrenal CS

In benign unilateral adrenal tumors, unilateral adrenalectomy is the first-line treatment (29). Medical treatment is very rarely indicated except when the patient's general medical condition does not allow surgery. Surgery is also considered the first-line treatment in patients with CS due to bilateral adrenal hyperplasia. Although the classical approach in bilateral disease was bilateral adrenalectomy, some studies showed that unilateral adrenalectomy could result in normalization of cortisol secretion (60% cases) or even AI (20% cases) (30). The major advantages of bilateral adrenalectomy are that there is no risk of recurrence if the resection is complete, and cortisol excess is always adequately controlled. Conversely, some studies have reported recurrence of hypercortisolism after unilateral adrenalectomy in 55% of patients with overt CS due to bilateral hyperplasia (31-35).

In the rare cases of ACC, mitotane has been shown to reduce the postsurgical recurrence rate when the resected cancer has a Ki67 proliferation index >10% (36). Surgery, of course, remains the first-line treatment whenever possible; but specific medical treatment for reducing cortisol concentrations should be used in combination with a systemic approach if cortisol concentrations are elevated in patients with metastatic disease. Although mitotane may be useful for controlling hypercortisolism, it requires weeks to achieve therapeutic levels, necessitating fast-acting agents in severe CS such as metyrapone or osilodrostat (6, 37).

## Available Medical Treatments

### Adrenal Steroidogenesis Inhibitors

**Figure 2.**
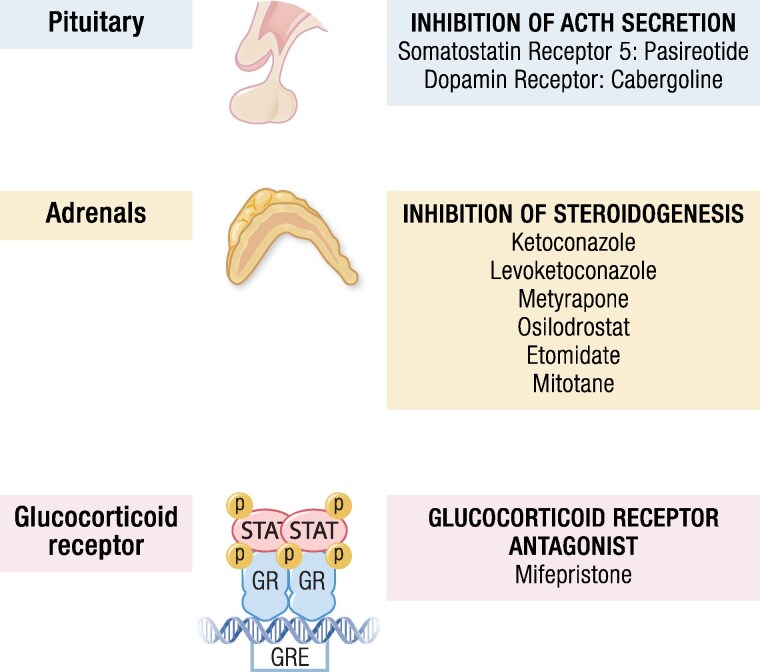
Mechanism of action of the available medical treatments for Cushing syndrome. Abbreviations: DRD2, dopamine receptor type 2; SSTR5, somatostatin receptor type 5.

To date, ketoconazole, levoketoconazole, metyrapone, osilodrostat, mitotane, and etomidate are the available drugs for use in patients with CS in clinical practice (Table 1) (9). They all block 1 or more enzymes involved in steroidogenesis (Fig. 2). This pharmacological group includes the most efficacious and widely administered medical therapies for the treatment of hypercortisolism and, for some of them, the most rapidly acting. Not all of them, however, are available worldwide.

**Table 1.** Summary of available medical treatments for Cushing syndrome treatment

Drug	Mechanism of action	Recommended dose	Efficacy	References
Ketoconazole	Inhibits CYP11A1, CYP17A1, CYP11B1, and B2	200 to 1200 mg/day	Normal UFC in 50% to 66%	Castinetti 2014 (Retr) (38) Viecceli 2022 (Retr) (39)
Levoketoconazole	Steroidogenesis inhibitor	300 to 1200 mg/day	Normal UFC in 50% to 81%	Fleseriu 2019 (ProspNC) (40) Pivonello 2022 (RCT) (41) Fleseriu 2022(ProspNC) (42)
Metyrapone	Inhibits CYP11B1 and B2	500 to 6000 mg/day	Normal UFC in 42% to 47%	Daniel 2015 (Retr) (43) Ceccato 2018 (ProspNC) (44) Nieman 2025 (ProspNC) (45)
Osilodrostat	Inhibits CYP11B1 and B2	2 to 60 mg/day	Normal UFC 77% to 83%	Pivonello 2020 (RCT) (46) Gadelha 2022 (RCT) (47)
Etomidate	Inhibits CYP11A1, CYP17A1, CYP11B1	0.1 mg/kg/h	Scarce data	
Mitotane	Steroidogenesis inhibitor	500 to 4000 mg/day	Normal UFC in 72%	Baudry 2012 (Retr) (48)
Pasireotide	SSTR agonist (with SSTR5 affinity)	1.2 to 1.8 mg/day	Normal UFC in 15% to 26%	Colao 2012 (RCT) (49)
Pasireotide LAR	SSTR agonist (with SSTR5 affinity)	10 to 30 mg/4 weeks	Normal UFC in 41% to 42%	Fleseriu 2019 (RCT) (50)
Cabergoline	Dopamine receptor subtype 2 (DRD2) agonist	0.1 to 7 mg/week	Normal UFC in 40%	Ferriere 2017 (Retr) (51)
Mifepristone	Glucocorticoid receptor competitive antagonist	300 to 1200 mg/day	60% improved hyperglycemia 88% improved Global Clinical Response	Fleseriu 2012 (RCT) (52)

Abbreviations: ProspNC, prospective noncontrolled study; RCT, randomized clinical trial; Retr, retrospective study; UFC, urinary free cortisol.

#### Ketoconazole

- **Mechanism of action:** Ketoconazole is a synthetic imidazole derivate that inhibits several enzymes responsible for cortisol synthesis, including cholesterol side-chain cleavage complex (*CYP11A1*), 17α-hydroxylase (*CYP17A1*), 11β-hydroxylase (*CYP11B1*), and aldosterone synthase (*CYP11B2*) (53). It has not been approved by the Food and Drug Administration (FDA), but it has the approval by the European Medicines Agency (EMA) for the treatment of CS in adults and adolescents older than age 12 years.
- **Efficacy:** In retrospective studies, 50% to 66% of patients achieved normal UFC concentrations after a mean treatment time of 4.05 ± 4.1 months (38, 39, 53). However, up to 25% of patients experienced treatment escape over time. For example, in the French multicenter FReSKO study with 200 patients with CD, at the last visit (mean period of 24.8 ± 33.6 months of treatment), 49.3% (n = 78) were controlled and 23.4% (n = 37) had partial control with at least 50% decrease of UFC (without normalization). However, only 33/51 patients (64.7%) treated for more than 24 months were controlled, whereas 12 (23.5%) had a partial response; interestingly, the other 6 patients (11.7%) had a final increase in UFC despite initial control after a mean time of 64.3 ± 27.3 (range 24.4-105.6) months (38). In general, no predictive factors of response to ketoconazole have been described (54), but in this study sex appeared to play a role because the proportion of males was higher in the uncontrolled than in the controlled group. This could be due to lower doses used because of hypoandrogenism. A meta-analysis including 35 articles with 1520 patients with different etiologies of CS reported a mean efficacy of 71% (95% CI, 52-88) for ketoconazole in normalizing cortisol concentrations (55). Another more recent meta-analysis demonstrated that control of hypercortisolism was achieved in 63% of patients (95% CI, 50-74) who received ketoconazole as a second-line treatment after transsphenoidal surgery (54). Of note, there is currently no prospective study available on ketoconazole use in CS.
- **Recommended doses:** Ketoconazole is orally administered at dosages of 200 to 1200 mg/day and due to its short half-life (3.3 hours), it requires a 2 or 3 daily administrations. The common starting dose is 200 to 600 mg/day. UFC normalization typically occurs at doses of 600 to 800 mg/day, but in cases of severe hypercortisolism, doses of up to 1200 mg/day may be necessary (38, 53). For adequate oral absorption, ketoconazole requires an acidic gastric environment. Even in experienced centers, up to one third of patients receive inadequate dose uptitration (38).
- **Safety:** The most common adverse events (AEs) include hepatotoxicity, gastrointestinal disturbances, AI, QT-interval prolongation, and skin rash. Hepatoxicity is one of the most concerning AEs associated with this drug. Because of reports of severe hepatotoxicity, the FDA and EMA have either suspended or released strong warnings regarding the use of ketoconazole as an antifungal agent. Nonetheless, the most common liver-related AE is mild or moderate increases in liver enzymes. For example, in the FReSKO study, mild (up to 5-fold normal values) and major (5- to 10-fold increase) elevations in liver enzymes were observed in 14% and 3% of patients, respectively (38). In this study, no cases of fatal hepatitis were observed. Moreover, since CS frequently causes fatty liver infiltration and increased liver transaminases, elevations of liver enzymes up to 2 to 3 times the ULN are not contraindications for use of ketoconazole but do require repeated careful monitoring. Androgen levels decrease with ketoconazole treatment, so acne and hirsutism associated with CS tend to improve in women, while men might develop gynecomastia and other hypogonadism-related symptoms. Ketoconazole is not recommended in pregnancy, being considered a Grade C recommendation according to the data of the FDA because of its teratogenic and embryotoxic effects and with the aim of preserving masculinization of the male fetus (56, 57). Drug-drug interactions are another important aspect that must be considered (Table 2).

#### Levoketoconazole

- **Mechanism of action:** Levoketoconazole is a 2S,4R stereoisomer of ketoconazole with a longer half-life and greater in vitro potency in inhibiting cortisol synthesis than ketoconazole (53). Levoketoconazole inhibits several enzymes in the steroidogenesis pathway in the adrenal cortex, including *CYP11A1, CYP17A1, CYP11B1,* and *CYP11B2* (61). Levoketoconazole has a similar mechanism of action to ketoconazole but differs in its higher inhibitory potency toward these CYP enzymes (61).
- **Efficacy:** In an open-label phase III study (SONICS study) with 94 patients with CS, 81% (62/77) achieved normal UFC at the end of titration phase (21 weeks) and 31% (29/94) had normal UFC at 6 months without further dose increase (40). In a more recent phase III randomized withdrawal study (LOGICS study), 50% of the patients achieved UFC control in comparation with 5% of the placebo group (41). In the extended evaluation of the SONICS study, which assessed the effects of levoketoconazole for an additional 6 months following a 6-month open-label maintenance phase, 61% exhibited normal mean UFC at month 6, 55% at month 9, and 41% at month 12 (42). Based on these findings, the FDA recently approved levoketoconazole for the treatment of CS in adults who are not surgical candidates or have experienced surgical failure, whereas the EMA gave an orphan drug indication. In relation to predictive factors of response, patients with diabetes mellitus and with lower UFC levels tend to respond better (40, 62). It is important to highlight that although the response rate was higher in patients with lower UFC concentrations at baseline, these patients required lower doses for UFC control. Thus, the differences in reported efficacy do not seem to be related to a failure of up-titration in the noncontrolled group. Moreover, levoketoconazole was effective in patients with severe hypercortisolism because UFC was normalized at the last maintenance phase assessment in 9 (56%) of 16 patients who had baseline UFC concentrations at least 5 times the ULN (40). While diabetes mellitus was suggested as a predictive factor of response, the reason of such association is not totally understood (62). Since then, some new cases treated with levoketoconazole have also been reported (63).
- **Recommended doses:** Levoketoconazole has a half-life of 4 to 6 hours and a 3-fold higher bioavailability than dextro-ketoconazole, allowing the administration of lower therapeutic doses (150-600 mg twice daily) (60) (Table 1). In clinical trials, the used doses range between 300 and 1200 mg/day (40-42). However, data on real-world use are still missing.
- **Safety:** The most frequent AEs are nausea, headache, and fatigue. Further AEs included peripheral edema, hypertension, diarrhea, and transaminitis (40-42). Regarding liver toxicity, levoketoconazole has a theoretical lower incidence of this AE compared to ketoconazole-induced liver toxicity, but the features overlap and usually manifest within the first 8 weeks of treatment as an idiosyncratic, mild-to-moderate, fully reversible transaminitis (61). QT-interval prolongation was described in 5% of the SONICS participants, whereas neither male hypogonadism nor unequivocal effects of levoketoconazole on testosterone secretion were reported in men (40). Although *CYP17A1*-mediated drug-drug interactions are less common with levoketoconazole than with ketoconazole, its 2-fold inhibition of *CYP3A4* may lead to interactions with substrates such as felodipine, atorvastatin, and testosterone (Table 2) (61). Overall, there do not appear to be major advantages to using levoketoconazole compared to ketoconazole.

#### Metyrapone

- **Mechanism of action:** Metyrapone is a pyridine derivative that inhibits 11β-hydroxylase and aldosterone synthase (64). Metyrapone is approved by the EMA for the treatment of CS, but its use is still off-label in the United States. However, it is approved for use as a test for AI by the FDA.
- **Efficacy:** A 12-week open-label prospective study (PROMPT study) conducted in 50 patients with CS, using a final median dosage of 1500 mg/day, showed that 47% of the patients treated with metyrapone normalized UFC and 80% showed a >50% decrease in UFC after 12 weeks; The efficacy was maintained at weeks 24 and 36 (45) A previous meta-analysis of retrospective data showed that metyrapone treatment was associated with biochemical remission in 76% of cases (95% CI, 57.5-90.9) (55). With the exception of the PROMPT study, evidence on the efficacy of metyrapone in the treatment of CS comes mainly from retrospective studies with heterogeneous designs (43, 44). For example, in the largest study on 195 patients with CS, metyrapone led to control of hypercortisolism in 43% of the patients after a mean follow-up of 8 months (43). Nevertheless, in a smaller study with 31 patients with CS but a similar follow-up period of 9 months, metyrapone induced remission in 70% (44). Although the efficacy of metyrapone seems to be lower than osilodrostat, a recent small uncontrolled study reported a similar reduction of UFC with both drugs, but osilodrostat was able to reduce cortisol levels and control blood pressure (BP) faster (65).
- **Recommended doses:** Metyrapone is orally administered at dosages of 500 to 6000 mg/day, but due to short half-life of only 2 hours, it requires multiple daily administrations up to 3 to 4 times a day. The median daily doses tend to be lower in patients with adrenal CS than in CD and in ECS (43). Metyrapone clearance is unaffected by renal or liver impairment until cirrhotic stage, but the concomitant use of some medications should be considered when making dose adjustments in clinical practice (Table 2) (60). A recent pilot study based on 15 patients suggested that the nadir cortisol obtained after a single dose of metyrapone could give insights on the total dose needed to control cortisol secretion (66).
- **Safety:** AEs are mainly mediated by the ACTH-driven accumulation of mineralocorticoid precursors (11-deoxycorticosterone) and androgen intermediates, leading to hypertension in a maximum of 48% of the patients (though this high rate was not reported in the largest retrospective study published (43)), peripheral edema in 3% to 23%, hypokalemia in 7% to 14%, and hirsutism or acne in 5% to 71% (55). These AEs are generally reversible with lowering of the metyrapone dose or specific management. In the PROMPT study, 6 participants developed reversible adrenal insufficiency during titration (45).

Metyrapone may be used in specific categories of patients, including children and the elderly. In children, long-term use of metyrapone as suppositories with clinical and biochemical improvement and no developmental side effects has been reported (67).

**Table 2.** Drug interactions with adrenal steroidogenesis inhibitors

Drug	Increase drug effect	Reduce drug effect
**Ketoconazole**	CYP3A4 inductors (rifampin, carbamazepine, phenytoin)	CYP3A4 inhibitors (itraconazole, clarithromycin, ritonavir) Avoid use of gastric acid suppressors (eg, H2-receptor antagonists and proton pump inhibitors)
**Levoketoconazole*^a^***	CYP3A4 inductors (rifampin, carbamazepine, phenytoin)	CYP3A4 Inhibitors (felodipine, atorvastatin, and testosterone) Avoid use of gastric acid suppressors (eg, H2-receptor antagonists, proton pump inhibitors)
**Metyrapone**	Drug-drug interactions are rare	Drug-drug interactions are rare
**Osilodrostat**	CYP3A4 and CYP2B6 inducers (58)	CYP3A4 inhibitors (58)
**Mitotane**	CYP3A4 inductors (see reference of Kroiss M, 2011 (59))	CYP3A4 inhibitors (see reference of Kroiss M, 2011 (59))

^*a*^Levoketoconazole inhibits adrenal steroidogenesis more potently than ketoconazole (relative half maximal inhibitory concentration = 1.2-2.7 against CYP11B2, CYP11A1, CYP17A1, and CYP11B1, respectively) (60).

#### Osilodrostat

- **Mechanism of action:** Osilodrostat is an imidazole derivate that inhibits 11β-hydroxylase *(CYP11B1)* and aldosterone synthase *(CYP11B2).* Thus, the mechanism of action is similar to metyrapone; however, osilodrostat might have a higher in vitro potency for inhibiting 11β-hydroxylase than metyrapone (64). In addition, a greater inhibition of the 17α-hydroxylase *(CYP17A1)* and 21α-hydroxylase *(CYP21A2)* has been reported with osilodrostat than with metyrapone; this may explain the lower increase in 11-deoxycortisol, androstenedione, and testosterone in females with the former (68, 69). Osilodrostat was approved by the FDA and the EMA in adults not cured by surgery or in whom surgery is not appropriate.
- **Efficacy:** The LINC trials evaluated the efficacy and safety of osilodrostat in patients with CD. The LINC-2 was a 22-week, open label, phase II trial of 19 patients with baseline UFC at least 1.5 above the ULN. In this study, osilodrostat led to normalization of UFC levels in 84% of patients at 10 weeks (79% at 22 weeks) (70). More robust data were provided by the LINC-3 study, a phase III, double-blind, randomized, withdrawal phase study that has included 137 patients with CD. In this clinical trial, the primary outcome was the proportion of patients with UFC normalization in the osilodrostat group vs placebo group at the end of the randomized withdrawal phase at week 34. After 12 weeks of open-label dose-titrated treatment and 12 additional weeks of open-label dose-optimized treatment, 72 (53%) patients had maintained normal UFC concentrations and were eligible for randomization. By week 34, at the end of the randomized treatment period, 31 (86%) of 36 randomly assigned to osilodrostat maintained normal UFC vs 10 (29%) of 35 randomly assigned to placebo (odds ratio [OR] 13.7 [95% CI, 3.7-53.4]) (46). Relevant data were also obtained in the extension phase of LINC3, which aims to evaluate the long-term efficacy of osilodrostat in patients with CD. It was observed that 81% of the patients who entered the extension phase (n = 86/106) had normal UFC at week 72 (71). The phase III study LINC 4, which included patients with biochemically milder CD in an upfront blinded placebo-controlled design, showed that osilodrostat caused normalization of UFC in 77% of the 48 patients randomized to osilodrostat compared to 8% of the 25 patients on placebo (47). In a recent pooled analysis of the LINC-3 and LINC-4 studies (210 patients, 48-week core phase, and open label extension), 50% of patients with increased systolic BP at baseline had normal systolic BP, and 61% with HbA1c > 6.5% had normal HbA1c at week 72, showing the efficacy of the drug to control hypertension and hyperglycemia (72). These improvements were more prominent in patients with both normal UFC and LSNC (73).

In addition, there is some evidence from real-world studies for the use of osilodrostat in patients with CD, ECS, and adrenal CS (27, 74, 75). For example, in the study by Dormoy et al (27) with 33 patients with severe CS due to an ECS, osilodrostat treatment led to control of hypercortisolism in 82% of cases when used as first-line monotherapy, 100% when used as second-line monotherapy, and in 68% in the group receiving combination therapy. In another series of 7 patients with ACC, osilodrostat resulted in normalization of UFC in all cases and in 6 patients normalization was achieved in the first 2 weeks of treatment (74). A recent real-world study (ILLUSTRATE) focusing on the efficacy of osilodrostat use in 42 patients with various etiologies of CS in the United States described normalization of UFC in 70% of the cases (75).

A comparative retrospective study of metyrapone (n = 6) and osilodrostat (n = 6) found that osilodrostat reduced UFC levels faster than metyrapone: after 2 weeks of treatment, UFC decreased by 21.3% with metyrapone vs 68.4% with osilodrostat (65). In this study, normalization of UFC was obtained in 42.9% of the cases treated with osilodrostat after 2 weeks of therapy and with a median dose of 6 mg/day, whereas no patient in the metyrapone group normalized UFC after 2 weeks of treatment. The median drug dose in the metyrapone group was 1000 mg at T1 (after 2 weeks), 1250 mg at T2 (4 weeks), and 1250 mg at T3 (12 weeks); for osilodrostat, the median dose was 4 mg at T1, 6 mg at T2, and 7 mg at T3. Nevertheless, these results should be interpreted with caution since this study provides no information about how the initial dose was chosen and how often doses were increased. Differences in UFC normalization could be related to dose and monitoring differences in both groups. Furthermore, similar rates of control for both drugs were seen at the end of the study at 12 weeks.

- **Recommended doses:** Osilodrostat is orally administered at dosages of 2 to 60 mg/day (76). Although osilodrostat has a higher potency than metyrapone and ketoconazole based on an experimental direct comparison in vitro (76), this has not been clearly shown in clinical practice. In addition, it has a longer half-life (4 hours), allowing a twice daily administration (77). Interestingly, Ferrari et al recently reported in 16 patients controlled with a stable dose of osilodrostat twice daily (7 Am and 7 Pm), that control of cortisol secretion could be maintained by switching the same total daily dose given once daily between 4 and 7 Pm (78). A pooled analysis of LINC studies also suggested the possibility of decreasing the dose over time for controlled patients (79). In classical nonsevere CS, osilodrostat is usually initiated at 2 mg, twice daily, orally, and titrated by 1 to 2 mg every 2 to 3 weeks based on cortisol levels and clinical parameters. In patients with very mild cortisol hypersecretion, it could also be initiated at 1 mg/day in the evening. A recent pharmacokinetic model demonstrated significant variability in osilodrostat responses among patients with ACTH-dependent CS. This variability should preclude the ability to define a precise initial dose based on hypercortisolism levels (80). However, in patients with severe hypercortisolism, higher starting doses should be considered. For example, in the Dormoy et al study (27), the median starting dose of osilodrostat was 10 mg/day (range, 2-40 mg/day) and the maximum dose reached 10 to 100 mg/day.
- **Safety:** The most frequent AEs are fatigue (29%-58%), nausea (32%-42%), headache (25%-34%), diarrhea (25%-32%) and, importantly, AI (46, 64, 70): hypoadrenalism-related adverse events were indeed reported in 51.1% of patients in the LINC3 study and 25% in the LINC-4 clinical trials. In these 2 trials, because of this side effect, osilodrostat was stopped permanently in about 3% of the patients, and transiently in 43% to 75% of the patients, with additional hydrocortisone being taken by 35% to 65% (46, 71). Interestingly, a recent series of cases suggested that some patients treated with osilodrostat for ACTH-dependent CS may experience adrenal gland shrinkage (46 ± 22.2%) with or without AI (81). Hirsutism and hyperandrogenism can also be seen in females. Osilodrostat-induced elevation in cortisol and aldosterone precursors can lead to hypokalemia, edema, and hypertension in some patients. The prolongation of the corrected QT interval has been observed in 4% of patients. An increase in ALT or AST was observed in 4% in the LINC 3 (46). Change of pituitary tumor size during treatment is infrequent. For example, in the LINC4, only 2 patients (2.7%) discontinued osilodrostat because of pituitary tumor enlargement; however, 40.0% had a ≥ 20% increase, but also 28.6% had a ≥ 20% decrease in tumor volume from baseline (47). Similarly, in the LINC3 trial, 4 of the 18 patients discontinued treatment because of pituitary tumor growth (46).

Because of the lack of data, currently, osilodrostat is contraindicated in pregnant women, children, and adolescents.

#### Etomidate

- **Mechanism of action:** Etomidate is a short-acting intravenous agent that inhibits adrenal steroidogenesis, primarily by blocking 11-beta-hydroxylase, but also CYP11A and CYP17A1 (82). It is considered an off-label treatment for CS by the EMA and FDA.
- **Efficacy:** Etomidate rapidly and profoundly decreases steroidogenesis within 12 to 24 hours, being reserved for cases of severe hypercortisolemia, especially where there are added complications such as respiratory failure or psychosis. In addition, because it is the only cortisol-lowering agent to be administered intravenously, it offers a unique alternative for patients who are unable to tolerate oral medications, and especially when intensive care is needed (83). A recent systematic review focused on the efficacy of etomidate for severe CS with a total of 36 articles comprising 76 clinical cases of 78 clinical episodes of severe CS. The time taken to achieve the lowest documented serum cortisol level ranged from 3 to 264 hours (median 38 hours, mean 60.22 hours) and, overall, 80.9% of patients survived long enough to receive definitive treatment (84).
- **Recommended doses:** Few data exist about adequate doses in CS. Nevertheless, based on previous case reports, it might be concluded that etomidate at a dose of 0.1 mg/kg/hour or lower is effective for the control of severe hypercortisolemia (85). Both ethyl alcohol etomidate and propylene glycol etomidate may be used (83). The standard protocol for administering etomidate in the intensive care unit to patients with severe and life-threatening CS includes an initial 5-mg bolus dose of etomidate over 2 to 3 minutes, followed by a continuous infusion starting at a rate of 0.02 mg/kg/hour that may be titrated (increase or decrease infusion rate in increments of 0.01 to 0.02 mg/kg/hour) no more frequently than every 6 hours based on measured serum cortisol concentrations (86, 87). According to this protocol, the recommended maximum infusion rate is 0.3 mg/kg/hour (86).
- **Safety:** Side effect of the most commonly available propylene glycol preparation vehicle are thrombophlebitis, pain on injection, and AI (83). Moreover, propylene glycol is associated with nephrotoxicity due to proximal renal tubular injury and lactic acidosis at high doses (88). Although etomidate is used in anesthesia to induce apnea and hypnosis in doses of 0.2  mg/kg to 0.4 mg/kg per hour, with the doses usually employed for hypercortisolism (0.04-0.05 mg/kg/hour), sedation is unfrequently induced (85). Due to high efficacy of this treatment, AI is very common, and in general a block and replace approach is recommended (83).

#### Mitotane

- **Mechanism of action:** Mitotane inhibits several steroidogenic enzymes and has a long-lasting adrenolytic action in steroid-secreting adrenocortical cells. Its use in patients with CS is usually reserved for patients with cortisol producing ACC (5).
- **Efficacy:** Mitotane suppresses hypercortisolism in 80% of cases, but with a slow onset of action and highly variable bioavailability and is not widely used outside the treatment of CS associated with ACC (89). In a series of 76 patients with CD treated with mitotane, at doses lower than used for cytotoxicity in ACC (leading to an effective theoretical threshold of blood mitotane superior to 8 mg/L compared to 14 in ACC), control was achieved in 48 (72%) of the 67 long-term treated patients, after a median time of 6.7 (5.2-8.2) months (48). In relation to ACC, although mitotane is effective in controlling hypercortisolism, its efficacy is delayed by several weeks. Thus, in general, for severe CS, other medical therapies with a more rapid effect are needed; but in mild hormone secretion due to ACC it may be enough for an effective control of hypercortisolism (6).
- **Recommended doses:** The recommended doses in patients with ACC (for antitumoral effect) range from 500 mg to 4 g total per day, orally (5). However, in patients with CD, remission was achieved with a mean daily dose of 2.6 ± 1.1 g (48). Mitotane should be initiated at rapidly increasing dose during the first 4 to 6 weeks, up to 2 to 4 g/day depending on the patient profile and UFC concentrations. Additionally, an initial monthly assay of plasma mitotane should be done for dose adjustment. Thus, monitoring of hypercortisolism should be performed using mainly UFC, as with other cortisol-lowering medications, but the periodically measurement of plasma mitotane concentrations is advisable especially to rule out overtreatment. In this regard, a negative relationship between plasma mitotane levels and UFC values has been described and all patients with a mitotane plasma concentration >8.5 mg/L had normal UFC concentrations (48). Mitotane is mainly used for patients with ACC, and in these patients 2 main regimens are implemented: a high-dose regimen, starting with 1.5 g/day and with a 1.5-g/day increase every day until achieving 6 g/day, followed by assessment of mitotane levels 2 to 3 weeks later; and the low-dose regimen, starting with 1.0 g/day and then increasing the doses by 0.5 g/day every 3 to 4 days up to 3.0 to 4.0 g/day, adjusting the dose based on mitotane levels and tolerability (6, 37). While the first approach may be especially useful in patients with ACC with good performance status, the latter may be suitable for patients with CD, that usually have lower cortisol levels than patients with ACC.
- **Safety:** AEs are very frequent with the use of mitotane (6), including gastrointestinal AEs such as nausea, vomiting, diarrhea and anorexia, hepatotoxicity, AI and central nervous system symptoms like lethargy, somnolence, vertigo, ataxia, confusion, depression, dizziness, and decreased memory. However, when used at lower doses than for ACC, intolerance leading to treatment discontinuation occurred less frequently (29% in patients with CD) (48). Nonetheless, similarly to patients with ACC, the most frequent AEs were gastrointestinal (47% of patients) and neurologic intolerance (30% of patients). Furthermore, mitotane has a high potential for drug-drug interactions and an inductive effect on several CYP enzymes (59, 90) (Table 2).

### Pituitary Tumor Directed Agents

#### Pasireotide

- **Mechanism of action:** Pasireotide is a multireceptor-targeted somatostatin receptor ligand with high binding affinity to 4 of the 5 known somatostatin receptor subtypes (SSTR1, SSTR2, SSTR3, and SSTR5). The main difference with octreotide and lanreotide is its high affinity for the SSTR5 and lower for SSTR2. Both receptors are expressed in many ACTH-secreting pituitary tumors, and those tumors harboring somatic *USP-8* mutations are more likely to have higher SSTR5 expression (91). Pasireotide is the first pituitary-directed agent to be approved for use in CD (92).
- **Efficacy:** Pasireotide was the first drug to be evaluated in large, prospective, randomized controlled trials and approved by regulatory agencies for CD, both in the daily subcutaneous and the intramuscular monthly long acting release (LAR) presentations. The first phase III clinical trial found a 15% and 26% UFC normalization with the 0.6 mg/12 hours subcutaneous dose and the 0.9 mg/12 hours subcutaneous dose, respectively (49, 93). These percentages were 42% and 41% for the LAR presentation at monthly doses of 10 and 30 mg, respectively, but the patients included in this study had milder disease than those included in the first subcutaneous dosing study (50, 94). Tumor volume reduction was seen in 56% and 47% of the patients included in these 2 clinical trials (49, 50). A good initial response was usually followed by long-term control; if biochemical control was not seen within 3 months of treatment initiation, it was unlikely to occur later and alternative treatment should be considered (49, 50, 95, 96). It should be noted that the median UFC level decreased by approximately 50% by month 2 and remained stable during the period of treatment in the phase III clinical trial (49). UFC concentration is a predictor of response: in the phase III clinical trial with pasireotide, UFC normalization was achieved more frequently in patients with baseline levels not exceeding 5 times the ULN (49). In addition, as mentioned previously, an in vitro study found that ACTH-secreting tumors harboring *USP-8* somatic mutations were more likely to be smaller and have higher SSTR5 expression; and thus, a better response to pasireotide could be expected (91).
- **Recommended doses:** The recommended initial dose for subcutaneous pasireotide is 0.6 mg twice per day. It can be increased to 0.9 mg twice per day. However, this form is rarely used since intramuscular pasireotide LAR became available. Pasireotide LAR should be initiated at 10 mg every 4 weeks in CD, titrating the dose up to 30 mg every 4weeks based on control of hypercortisolism and tolerability. Unlike steroidogenesis inhibitors, higher doses do not necessarily mean higher efficacy. Increasing the dose to 40 mg has not shown any clear benefit. Lower subcutaneous doses (150-360 µg twice daily) have shown effectiveness in some cases, causing less hyperglycemia than the standard dosage (97, 98) (Table 1). This also suggests that a downtitration could be performed once the disease is under control with pasireotide LAR, especially in patients with diabetes.
- **Safety:** Hyperglycemia occurs in around 75% of patients receiving pasireotide (49, 50, 99), with it being more common with higher age and previous glycemia. Associated hyperglycemia can be significant and may necessitate treatment withdrawal, together with multimodal stepwise management using a variety of antiglycemic agents and insulin/insulin analogues but does reverse after pasireotide withdrawal. Other common AEs are diarrhea, cholelithiasis, and nausea (49, 50, 96).

#### Cabergoline

- **Mechanism of action:** Cabergoline is a highly selective agonist for the dopamine receptor subtype 2 (DRD2), which is expressed in many ACTH-secreting pituitary tumors. DRD2 expression has been clearly demonstrated either at messenger or at protein levels in humans with corticotroph pituitary tumors in variable percentages between 37.5% and 83.3% and between 20% and 89.4%, respectively, according to the different techniques used for receptor detection (100, 101).
- **Efficacy:** Cabergoline may be used off-label as a treatment of hypercortisolism in CD. It has only been evaluated in small retrospective noncontrolled studies with a limited number of patients (51, 102, 103). The largest multicenter study included 53 patients and showed UFC normalization in 40% of them, but only 21% control in the long term (more than 12 months) (51), due to the known escape phenomenon that occurs in many patients (∼22%) treated with cabergoline (101). Individual cases have also shown a reduction of tumor volume (102, 103). Predictive factors to identify patients who will respond to cabergoline treatment have not been found.
- **Recommended doses:** Cabergoline is administered orally, usually once to twice per week, considering its long half-life. Administered doses for CD are usually higher than those more commonly used for patients with hyperprolactinemia and range from 0.5 to 7 mg/week (101-103). For example, in the largest study published (51), the mean cabergoline dosage associated with UFC normalization in complete responders was 1.5 mg/week (range: 0.5-4.0), but the dose needed was <2, 2 to 3.5, and ≥3.5 mg in, respectively, 52%, 24%, and 24% of patients (Table 1).
- **Safety:** Common AEs are nausea, dizziness, and postural hypotension. Psychiatric side effects (mainly impulse control disorders) are less common, but patients should be warned about these important issues. While cardiac valvulopathy has been described in patients with Parkinson disease treated by ergot dopamine agonists, this risk is still debated in prolactinoma as cabergoline is usually employed at lower doses. In CD, as the dose used might be higher and maintained on a long-term basis, it seems reasonable to consider that an echocardiogram should be performed at treatment initiation, and then, depending on the dose, every 2 to 5 years (101-103). Cabergoline may be an effective and safe therapeutic option for the treatment of CD during pregnancy, especially in cases of mild hypercortisolism (104, 105).

#### Glucocorticoid receptor antagonist: mifepristone

- **Mechanism of action:** Mifepristone is a competitive antagonist of the glucocorticoid and the progesterone receptors, and an androgen antagonist. It is also an inhibitor of CYP3A4 and CYP2C8/9. Therefore, it is essential to consider potential drug-drug interactions (106, 107). It is approved by the FDA for the treatment of hyperglycemia secondary to hypercortisolism in patients with CS who have failed surgery or are not candidates for surgery. Elevated cortisol concentrations that can occur due to mifepristone's mechanism of action can saturate the binding capacity of 11β-hydroxysteroid dehydrogenase type 2, leading to increased availability of cortisol to stimulate the mineralocorticoid receptor (106).
- **Efficacy:** In a 24-week open-label, multicenter study (SEISMIC trial) including 50 participants with endogenous CS and hypertension or hyperglycemia, 60% of the patients with hyperglycemia and 38% of those with hypertension fulfilled the prespecified response criteria. Significant improvement was also observed in weight, waist circumference, insulin resistance, depression, cognition, and quality of life (52, 108). Both corticotrophin tumor progression and regression have been described with mifepristone treatment, without correlation with ACTH levels (109). The increase in ACTH concentrations after treatment has been described in both ACTH-dependent and ACTH-independent CS (110, 111). Mifepristone has also demonstrated significant improvement in psychotic symptoms, hypertension, and hyperglycemia in patients with ECS at a median dose of 600 mg/day (112). Data on long-term efficacy and safety (beyond 24 months) are scarce (113).
- **Recommended doses:** The usual dose ranges from 300 to 1200 mg/day. ACTH and cortisol usually increase due to the glucocorticoid receptor blockade, hindering dose adjustment, which can only rely on clinical parameters (Table 1).
- **Safety:** Common AEs are fatigue, nausea, vomiting, headache, dizziness, arthralgia, (resembling AI symptoms), and endometrial thickening in women. In the SEISMIC trial, most AEs improved after the first weeks of treatment despite dose increase. High levels of serum cortisol can induce hypertension, edema, and hypokalemia due to cortisol binding to the mineralocorticoid receptor (52, 114). Hypokalemia can be anticipated and prevented through close monitoring and judicious use of potassium supplements and spironolactone. Other recommendations aimed at minimizing AEs are to adjust the antidiabetic treatment to reduce the risk of hypoglycemia, monitoring of blood pressure and adjustment of antihypertensive drugs, warfarin dose reduction (due to drug-drug interaction), avoidance of drugs that prolong the QT interval (since mifepristone prolongs the QT interval in a dose-related manner (106)), and planning of periodic mifepristone holidays in women, combined with a course of medroxyprogesterone to induce decidual bleeding (106). In relation to AI, it is important to highlight that cortisol levels are not useful to monitor adrenocortical function while titrating the mifepristone dose; thus, it is important to monitor clinically for signs of AI. In case of AI development, treatment should be initiated promptly with potent high-dose glucocorticoids to overcome the glucocorticoid receptor-blocking effect of mifepristone and mifepristone should be discontinued (115).

### Combined Treatment

Combinations of medical treatments can be used off label for controlling hypercortisolism. In general, the combination of drugs with different mechanisms of action improves the efficacy of single drugs because of the additional or synergic actions, allowing a reduction of the doses for each drug, and therefore potentially reducing the incidence of AEs (53). This approach may be particularly useful in the short-term while awaiting surgery and in patients with severe hypercortisolism. Nonetheless, there are limited data based mainly on small retrospective studies.

- **Ketoconazole and metyrapone:** The combination of both drugs has been successfully used in cases of severe CS. For example, in a retrospective study of 14 patients with ECS and 8 with ACC with severe hypercortisolism, after 1 week of treatment, median UFC decreased from 40.0 to 3.2 ULN in patients with ECS and from 16.0 to 1.0 ULN in patients with ACC. In addition, after 1 month of treatment, UFC values were normal in 73% and 86% of patients with ECS and ACC, respectively (26). In another study, the preoperative treatment with the combination of ketoconazole and metyrapone in 22 patients with CS achieved normal UFC in 45% of the cases at a median of 4 months (116).
- **Ketoconazole, metyrapone and mitotane:** This combination was employed in a follow-up study of 11 patients with severe CS. High-dose therapy combining mitotane (3.0-5.0 g/24 hours), metyrapone (3.0-4.5 g/24 hours), and ketoconazole (400-1200 mg/24 hours) was initiated concomitantly in all cases, and this regimen led to control of hypercortisolism in 64% and an improvement of the clinical syndrome and comorbidities in most of the cases. However, AEs were common: hypokalemia in 100%, increase in liver enzymes in 18.2% to 81.8%, nausea and vomiting in 64%, and AI in 36% (25).
- **Ketoconazole and cabergoline:** In a prospective study of 14 patients with CD, the combination of ketoconazole and cabergoline led to UFC normalization in 79% of patients (117). Another study focused on comparing the efficacy of cabergoline in monotherapy and in combination with ketoconazole: after a mean follow-up of 6 months, normalization of UFC was reported in 25% of the patients treated with cabergoline (2-3 mg/week) used as monotherapy. The addition of ketoconazole to the 9 patients without an adequate response to cabergoline was able to normalize UFC excretion in 67% of the cases (118). AEs were uncommon, with a mild increase in liver enzymes only reported in 11% of the patients.
- **Other combinations:**
  - The combination of osilodrostat and etomidate has been successfully employed in a case of severe ACTH-dependent CS in a patient initially unresponsive to osilodrostat (119). Treatment with etomidate was discontinued on the 19th day of the combined therapy and then the osilodrostat doses were reduced and proper control of UFC was achieved.
  - In the largest study on ECS treated with osilodrostat (27), osilodrostat was used in combination with ketoconazole in 6 patients (although both drugs may increase QTc), with ketoconazole and metyrapone in 1 patient, with cabergoline in 1 case and with metyrapone in another. Biochemical control of CS was achieved in 67% of the cases and no severe AEs were observed. Precise details were not provided.
  - Combination of pasireotide at dosages of 300 to 750 μg/day, cabergoline at dosages of 2 to 6 mg/week, and ketoconazole at a dosage of 600 mg/day resulted in control of CD in 7 of 8 patients. It was reported using a stepwise approach with pasireotide as the starting treatment and cabergoline and ketoconazole as first and second additional treatment (120). In this regard, it should be noted that simultaneous targeting of SSTR5 and D2 for a corticotroph adenoma could result in synergistic inhibitory effects (121). Supporting the combination of cabergoline and pasireotide, a multicenter study (CAPACITY trial, NCT01915303) of 66 patients with CD showed that addition of cabergoline to pasireotide doubled the number of patients with controlled disease to almost 40% of the study population (122). In this study, 27 patients received pasireotide monotherapy and 39 received combination therapy. Overall, 37.9% (n = 25) of the cases achieved the primary endpoint (patients with median UFC below the ULN at week 35). Of these 25 patients, 13 received pasireotide monotherapy, with a further 12 patients achieving median UFC ≤ ULN at week 35 following the addition of cabergoline.
  - Finally, a recent study discussed the benefits of a combined approach with ketoconazole induction therapy followed by octreotide in 14 patients: 11 had normal UFC while on ketoconazole and 3 remained controlled (4 had a partial response) when switched to octreotide (123).

## Perspectives on New Potential Treatments

**Figure 3.**
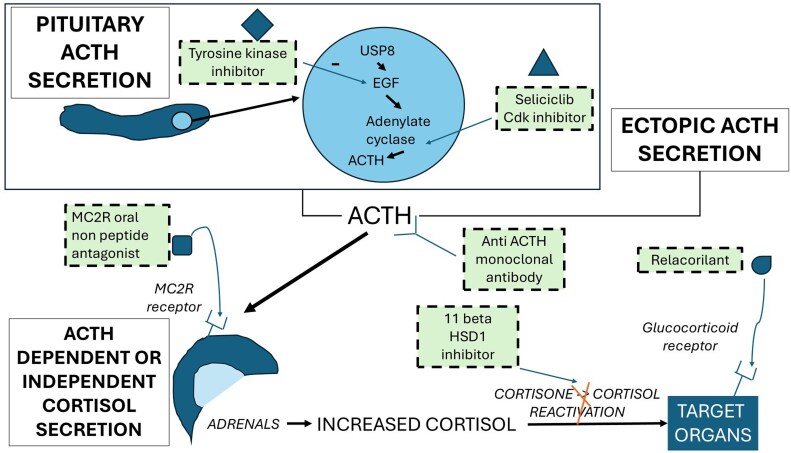
Perspectives in the medical treatment of Cushing disease. Abbreviations: Cdk, cyclin-dependent kinase; EGF, epidermal growth factor; USP8, ubiquitin-specific peptidase 8.

The therapeutic armamentarium for CS is now rich with 3 different mechanisms of action: steroidogenesis inhibitors that reduce cortisol at the adrenal level, drugs that target the pituitary either through dopamine or somatostatin receptors, and drugs that target the glucocorticoid receptor to avoid the effects of excess cortisol. However, some patients remain uncontrolled or experience side effects from the various medications. New potential treatments target pituitary signaling pathways, ACTH or its adrenal receptor, or the regeneration of cortisol in peripheral tissues (Fig. 3).

**Relacorilant:** Relacorilant is a selective modulator of the glucocorticoid receptor that antagonizes cortisol activity without binding to the progesterone receptor (124). In this regard, given its binding affinity profile, it was hypothesized that relacorilant would provide the benefits of cortisol modulation, but without the unwanted effects of progesterone receptor antagonism in the treatment of patients with CS (125). Relacorilant has been evaluated in a phase II, multicenter, open-label study, including 35 patients with CS and uncontrolled hypertension or hyperglycemia, with 2 dose groups. The primary outcomes were a clinically meaningful improvement in systolic or diastolic BP or in diabetes control. These outcomes were achieved by 5/12 and 7/11 for hypertension (HTN) and by 2/13 and 6/12 for hyperglycemia (diabetes mellitus (DM)/impaired glucose tolerance (IGT)) in the low-dose (100-200 mg/day) and high-dose (250-400 mg/day) groups, respectively (125). Parts of the results of GRACE, a 2-part phase III study of relacorilant in patients with CS of all etiologies and uncontrolled hypertension and/or hyperglycemia, were recently reported in abstract form (126). In this 22-week open-label phase followed by a 12-week, double-blind, placebo-controlled randomized withdrawal phase, 152 patients (n = 31 with HTN; n = 50 with DM/IGT; n = 71 with both) were enrolled in the open-label phase and received relacorilant 100 mg once daily titrated up to 400 mg as tolerated. At their last assessment at week 22, 63% of patients achieved HTN and/or DM/IGT control. The recommended doses ranged from 100 to 400 mg/day, but more studies are needed to establish the dose with the best risk-benefit ratio. For mifepristone, dose adjustment is not straightforward and has to rely on clinical parameters, since ACTH and cortisol levels increase in response to cortisol receptor antagonism, although to a lesser extent than with mifepristone (Table 1). AEs in the study included back pain, headache, peripheral edema, myopathy, nausea, diarrhea, and dizziness, and were more common in the high-dose group, but because there was no placebo group, it is difficult to know to what extent they are the result of the drug. Vaginal bleeding, hypokalemia, and worsening hypertension did not occur. Although no cases of AI were reported in the phase II clinical trial of relacorilant (125), due to its mechanism of action, the same considerations for AI than with mifepristone should be applied. Of note, there is an ongoing pivotal phase III trial of relacorilant in patients with CS (GRACE trial) (NCT03697109). The primary outcome is the achievement of sustained BP control during the randomized-withdrawal phase, wherein patients who had achieved the BP response criteria during the open-label phase are randomized to receive either relacorilant or placebo for 12 weeks.

**Seliciclib (roscovitine) is** a small-molecule, selective cyclin-dependent kinase inhibitor, which inhibits cell proliferation, *POMC* transcription, and ACTH production in neoplastic corticotrophs in vitro. Melmed's team had previously deciphered some mechanisms of corticotroph cells proliferation: *PTTG* overexpression, induced by E2F1, leads to cyclin E upregulation, and this is involved in uncontrolled *POMC* transcription and ACTH production (127). In a recent proof-of-concept single-center open label phase II clinical trial, Liu et al (128) reported the efficacy and safety of seliciclib, given 400 mg twice daily in 9 females with de novo or persistent or recurrent CD, including 5 who had been previously treated by ketoconazole, pasireotide, or cabergoline. Initial 24-hour UFC ranged from 98 to 368 µg/24 hours (ULN, 50 µg/24 hours). After 4 weeks of treatment, UFC was reduced by 42% (range, 11%-75%, decrease was seen in all patients, but none reached a normal UFC level): in 3 patients, UFC was reduced by >50%, associated with a 19% reduction in ACTH. The maximal effect was seen at week 1. Of note, grade 4 liver toxicity was reported in 2 patients, which resolved after 4 weeks of withdrawal. Seven patients thus completed the 4-week period of treatment. Because of this liver toxicity, a new open-label phase II study should be launched with a lower initiation dose of the compound, as preclinical data suggested that side effects were dose-dependent: this will allow determine the lowest effective dose of seliciclib.

Small molecules targeting ACTH (**anti-ACTH monoclonal antibody,** LuAg13909, Lundbeck) or its receptor (**once-daily oral nonpeptide MC2R antagonist**, CRN04894, Atumelnant, Crinetics) are also promising. Interestingly, these drugs are also being investigated as potential treatments for classic congenital adrenal hyperplasia in the aim of decreasing ACTH levels to decrease androgens. Results of a phase IB/II study with atumelnant (NCT05804669) were recently reported, with normal UFC achieved in 5 patients with ACTH-dependent CS treated for 10 days with 80 mg atumelnant. Serum cortisol was < 5 µg/dL in all patients after 10 days, emphasizing the rapid efficacy of this drug and the potential risk of AI, which will need careful monitoring. No specific side effect was reported (129). LuAg13909, which targets ACTH synthesis, recently entered a phase II trial in patients with ACTH dependent CS.

Another approach may be to reduce cortisol production from cortisone in peripheral tissues. 11-beta hydroxysteroid dehydrogenase type 1 (11beta-HSD1) converts inactive cortisone to active cortisol. **SPI-62 (clofutriben)** is an inhibitor of 11beta-HSD1 thus blocking the conversion of cortisone to cortisol. Interestingly, Tomlinson et al (130) had reported more than 20 years ago the case of a 20-year-old man, presenting with an obvious biochemical hypercortisolism (UFC > 3-fold the ULN), but no clinical sign because of a partial defect in 11beta-HSD1. The drug is currently being evaluated in a multicenter, randomized, placebo-controlled study in patients with ACTH-dependent CS (NCT05307328). Patients will receive SPI-62 or a placebo for a total of 24 weeks, with the option of long-term extension.

Finally, a major pathophysiological advance over the past 10 years was the discovery of *USP8* somatic mutations as a driver of tumorigenesis in half of ACTH-secreting pituitary tumors. These gain-of-function mutations alter EGFR pathways by reducing EGFR ubiquitination, leading to sustained epidermal growth factor (EGF) signaling, and overexpression of EGF receptors. **Tyrosine kinase inhibitors targeting EGF-R** could be potential new candidates as a treatment of CD: while in vitro studies suggested promising results, no clinical trial has seemingly shown efficacy up to now. As detailed in a recent review (131), **gefitinib** (approved in metastatic non-small-cell lung cancer), an oral small molecule EGFR inhibitor, is currently under evaluation in patients with USP8-mutated CD (NCT02484755). USP8 inhibitors could also be promising, as they decrease *POMC* transcription, ACTH levels, and cell proliferation in vitro (132). Finally, *BRAF V600E* mutation has been identified in 0% to 16% cases of CD without USP8 mutation. **Vemurafenib**, a small BRAF kinase inhibitor, has also shown promising results in vitro with suppressed ACTH secretion without decreasing cell viability (131, 133).

## Titration and Block-and-Replace Strategies

**Figure 4.**
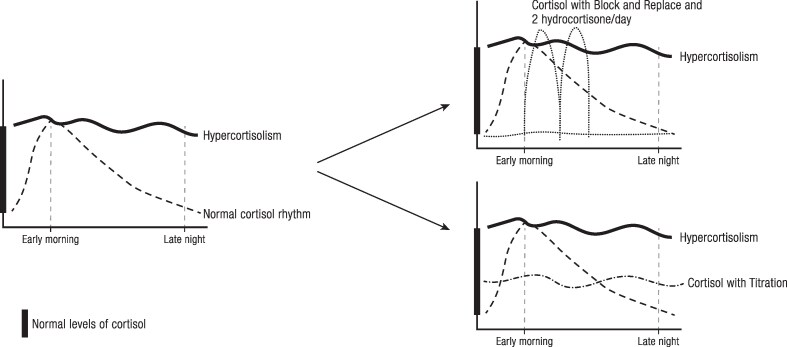
Block and replace vs titration approach. Left panel: The thin dashed line represents normal cortisol levels throughout a 24-hour day; the bold solid line shows cortisol levels in Cushing syndrome. Right panel, upper section: Block-and-replace approach allows cortisol levels to rise during the day and drop at night by using two hydrocortisone doses per day (indicated by peaks in the thin dotted line). Right panel, lower section: Titration approach allows for an overall normal level of cortisol stable throughout the day (shown by a thin dash-dotted line). The bold area on the Y-axis represents the range of normal levels of cortisol.

**Figure 5.**
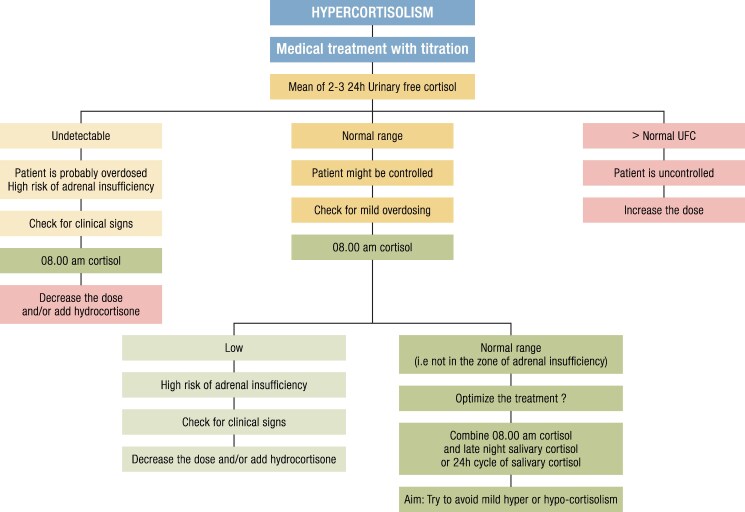
Monitoring of treatment efficacy using the titration regimen (adapted from Castinetti F, 2022 (140)). Abbreviations: LNSC, late-night salivary cortisol; UFC, urinary free cortisol.

Treatment with steroidogenesis inhibitors can be initiated in 1 of 2 ways: a titration or a block-and-replace approach. Titration requires adjusting the daily drug dose to normalize cortisol concentrations. Alternatively, the block-and-replace approach uses a higher drug dose to fully suppress cortisol (ie, induce AI), followed by cortisol supplementation (134) (Fig. 4). Both strategies have their pros and cons and should be tailored to different patient profiles based on the severity of hypercortisolism and the fluctuations of underlying cortisol excess. In general, block and replace may be favored in severe disease, where there is evidence of cyclicity in cortisol secretion, or where monitoring is less straightforward, as happened during the COVID pandemic (135). For example, Dormoy et al (27) described an algorithm with osilodrostat to choose between titration and block-and-replace regimens in patients with ECS based on the intensity of hypercortisolism, the associated complications, and the tumor status of the patients. Although the titration regimen involves a gradual dose increase, thereby possibly causing fewer side effects and lower overall doses of medication, there is a risk of both overtreatment leading to life-threatening AI (caused by excessively reduction in cortisol synthesis and/or action its receptor) as well as undertreatment resulting in inadequately controlled cortisol excess (5, 134). Of note, it may be difficult to distinguish AI from glucocorticoid withdrawal because the symptoms may overlap (58). Regular clinical and biochemical monitoring for treatment efficacy and AEs is therefore required in all patients receiving titration therapy (136). Most studies of steroidogenesis inhibitors have used UFC as the primary marker to evaluate antisecretory efficacy (38, 40, 43, 46). However, given its high intrapatient variability during repeated sampling (137), collecting 2 to 3 UFC samples may be required to improve reliability of dose adjustments. Serum cortisol day curves offer another, though cumbersome, alternative (138). In addition, morning serum cortisol should be measured regularly with a low value (<5 µg/dL) indicating AI, and a target value of 8 to 10 µg/dL possibly indicating good biochemical control (136). Another marker that can be used to assess drug efficacy is LNSC. LNSC is easy to collect, enables long-distance surveillance, and has the advantage of evaluating the nadir of cortisol. In the phase III study of pasireotide LAR, optimal metabolic profiles were observed in patients with both normal mean UFC and LNSC (73, 139). Nevertheless, the correlation between LNSC and UFC was moderate (Spearman correlation: ρ = 0.50) and no information on baseline serum cortisol correlation was described in this study (139). In the pooled data from 2 phase III osilodrostat studies (LINC3 and LINC4), at week 72, 48.6% of patients had both LNSC and UFC controlled; these patients had a greater improvement in cardiovascular/metabolic-related parameters and quality of life than those with only UFC controlled or both LNSC and UFC uncontrolled. One important finding of this study was that despite lower morning serum cortisol in patients with both normal UFC and LNSC than in those with high LNSC, no patients had morning cortisol levels below the lower limit of normality (73). This combined approach might however increase the risk of inducing AI in patients with minimal cortisol diurnal variation (Fig. 5) (140).

Finally, another important point to consider when patients are treated by metyrapone or osilodrostat is the lack of reliability of the immunoassay measurement of cortisol because of the cross-reactivity between cortisol and 11-deoxycortisol, leading to an overestimation of the former in some (older) immunoassays. Thus, serum and urinary cortisol should be measured using tandem mass spectrometry or immunoassays employing highly cortisol-specific monoclonal antibodies in patients treated with metyrapone or osilodrostat (141, 142).

In addition, the Haircush Study showed that despite normalized UFC levels, medically treated patients with CD had an altered circadian rhythm of serum cortisol. The authors proposed that a single hair-cortisone measurement identifies chronic mild persistent hypercortisolism and could replace multiple saliva analyzes to monitor medical treatments in CD patients once UFC is normalized (143).

Because all biomarkers provide different information, we suggest that the optimal treatment monitoring approach probably involves a combination of them: UFC to estimate overall secretion, LNSC to assess cortisol nadir (both parameters test for underdosing), and morning serum cortisol to detect overdosing (Fig. 5) (136, 140). Another interesting tool to identify adrenal insufficiency/sufficiency that allows remote monitoring is morning salivary cortisone measurement (144). In a prospective study enrolling 220 patients, with a prevalence of AI (diagnoses based on ACTH stimulation test) of 44%, the area under the receiver-operating characteristic curve for waking salivary cortisone as a predictor of AI was 0.95 (95% CI, 0.92-0.97) (144).

The block-and-replace approach can be used off label for all types of steroidogenesis inhibitors, although it is most commonly used for metyrapone (alone or in combination with ketoconazole) and osilodrostat. Some rare cases have been reported with ketoconazole used as monotherapy in a block-and-replace regime (38). Once established, the block-and-replace strategy does not require as frequent biochemical controls as the titration approach. However, it is important to ensure that adequate blockade is achieved, to avoid perseverance of the cushingoid state due to incomplete blockade and exogenous glucocorticoid substitution (136, 140). The required doses of steroidogenesis inhibitors may vary greatly between patients, and inhibition of UFC and morning serum cortisol should be documented on several occasions. For glucocorticoid replacement, weight-adjusted standard doses of hydrocortisone that are used in AI are recommended, typically 15 to 20 mg/day divided into 2 to 3 doses, or prednisolone at doses around 3 to 5 mg daily (136). Although these are the usual recommended doses, they may vary according to the body surface area (145). In severe hypercortisolemia when initiating either high doses of steroidogenesis inhibitors or using highly potent steroidogenesis inhibitors (osilodrostat) in a block-and-replace fashion, it is not unreasonable to initiate glucocorticoid replacement therapy at the same time. This will reduce the risk of AI while only adding a small extra glucocorticoid burden. It is then important to assess after days and weeks that a full blockade has been achieved in order to avoid excessive exogenous glucocorticoid administration in case the medical treatment was not immediately effective. For the assessment of full blockade, following administration of the morning dose of hydrocortisone, the afternoon dose is omitted, and serum cortisol is measured the next morning (ie, 24 hours after the last hydrocortisone intake). If UFC assessment is required, urine collection should begin at that time, and hydrocortisone administration should be delayed until the collection is complete. Other glucocorticoids cause less interference with cortisol measurements in serum and urine. Another option is to start the treatment with no replacement and add hydrocortisone when morning serum cortisol concentrations reached 10 to 11 µg/dL (27).

## Treatment of Hypercortisolism in Special Situations

### Cyclic CS

Certain aspects of CS require special attention, with 1 of the most challenging scenarios being cyclic CS (cCS). This condition is characterized by alternating phases of biochemical hypercortisolism (peaks) and periods of physiological or even hypocortisolemic cortisol concentrations (troughs) (146). Currently, there is no standardized definition of cCS, and various alternative terms (eg, variable, intermitting, episodic) have been proposed to describe those strong fluctuations in cortisol secretion (147). Identifying at least 2 peaks and 1 trough in cortisol concentration seems to be a reasonable approach for detecting true cyclicity while avoiding excessively long observation periods (146, 148). The largest retrospective study on cCS based on 110 patients gave insights into epidemiology: 64% of the cases were pituitary, 23% were ectopic, 3% were adrenal, and 11% were occult (149). Treatment for cCS generally aligns with the management strategies used for “noncyclic” CS. However, misleading biochemical test results can delay or impede correct diagnosis. In addition, because both ectopic and occult tumor origins are also possible in cCS, medical therapy is frequently necessary to manage the condition while awaiting accurate tumor localization (146). Three important aspects need to be considered when initiating medical therapy in a patient with suspected or confirmed cCS:

- The unpredictable intensity of cortisol excess
- The unpredictable duration of peak and trough phases
- The potential of spontaneous phases of hypocortisolism which could lead to life-threatening adrenal crises: adrenal insufficiency was indeed reported in 28% of cases in the largest retrospective series on cCS (149).

Given these challenges, a block-and-replace approach is generally recommended in patients with cCS (5). As always with block-and-replace therapy, high doses of steroidogenesis inhibitors may be necessary to effectively suppress endogenous cortisol production and patients should be carefully instructed on when and how to adjust their hydrocortisone replacement therapy. Clinical parameters such as improvements in BP, body weight, potassium levels, and blood glucose can serve as additional indicators of adequate cortisol control. Since spontaneous remission phases lasting several years have been reported (146), it may be reasonable to consider pausing block-and-replace therapy in patients who have maintained stable doses and achieved good clinical control over an extended period. However, close clinical and biochemical monitoring is crucial to detect early signs of relapse. Patients suspected of having cCS should be referred to centers with expertise in both cCS and medical therapy to minimize complications arising from the diagnostic and therapeutic challenges associated with this condition.

### Pregnancy

The coincidence of pregnancy and CS is an infrequent and challenging situation. Although hypercortisolism inhibits the secretion of gonadotropins, reducing fertility, both CD and cortisol-producing adrenal adenomas tend to present in women of childbearing age, so this coincidence can occur, and about 300 cases have been described in the literature. Hypercortisolism worsens the prognosis of pregnancy, as it is associated with preeclampsia, gestational diabetes, and increased maternal and fetal morbidity (150-155). Thus, women of childbearing age need contraception during active CS (preferably without using estrogens, due to the risk of thrombosis), and particularly when receiving pharmacological treatment, as restoration of eucortisolism might allow for normal ovulation.

There are not enough studies to provide evidence-based management of CS during pregnancy, so treatment must be individualized based on the cause, timing of diagnosis, and severity of hypercortisolism. Experts recommend managing milder cases with symptomatic treatment of comorbidities (diabetes, hypertension), considering thromboprophylaxis with low-molecular-weight heparin, and delaying definitive treatment until after delivery. More severe cases should be referred to for surgical treatment (pituitary or more frequently adrenal) in the second trimester, before 24 weeks of gestation. However, some women will need pharmacological treatment for hypercortisolism, at least during some periods of their pregnancy, either due to contraindication, patient refusal, or high risk of surgical treatment, or as treatment of severe hypercortisolism as a preparation for surgery or during the first and third trimesters (150, 153-155). In women with CS on chronic pharmacological treatment who desire pregnancy, the different available options, including bilateral adrenalectomy, should be discussed with the patient before pregnancy. In relation to the selection of active treatment of hypercortisolism with surgery or medical treatment vs conservative management (only treatment of hypercortisolism-related comorbidities), a systematic review demonstrated that active treatment of hypercortisolism through surgery or drugs reduced fetal losses but no other complications such as prematurity, intrauterine growth retardation, or stillbirth (152).

When the use of drugs is considered indicated, it must be noted that none is approved by regulatory agencies for use in pregnant women, and most of the information available comes from published case reports. However, metyrapone and cabergoline are considered the best options, and there are several published cases of successful pregnancies in women treated with them. Treatment monitoring will be based on clinical parameters, such as weight and blood pressure and biochemical parameters (e.g., glucose, sodium, potassium). Regarding cortisol levels, it is essential to remember the changes that the hypothalamic-pituitary-adrenal axis undergoes during pregnancy (increased serum cortisol (total) due to higher hepatic production of corticosteroid-binding globulin, increased UFC due to placental CRH release, reduced cortisol-induced negative feedback because of the antiglucocorticoid effect of progesterone), and aim for serum cortisol concentrations of 2 to 3× ULN and UFC concentrations around 1.5× ULN (5, 136, 150, 155). This threshold is justified because during pregnancy the rise in corticosteroid-binding globulin can be up to 3 times the normal value during the third trimester, leading to 2- to 3-fold increase of plasma cortisol levels. UFC also increases during the second trimester (1.4- to 1.6-fold increase in the second and third trimester, respectively).

There is less information about nocturnal salivary cortisol during pregnancy. It is higher than in nonpregnant women but retains the circadian rhythmicity and could therefore be a useful parameter in adjusting drug doses in this context (156).

Metyrapone is the most commonly used drug and has been shown to be effective and safe in published clinical cases (56, 156-162). It has been used at doses of 500 to 3000 mg/day and has the advantage of rapid onset of action. However, it can worsen BP from the accumulation of deoxycorticosterone and cross the placental barrier, impairing fetal steroidogenesis. Ketoconazole is considered a less safe option, as it could cause feminization of male fetuses due to its inhibitory effect on androgen synthesis, especially if used during the first trimester of pregnancy. It has also shown teratogenicity and increased rate of abortion in animal studies (150, 153). However, there are also a small number of clinical cases where it has been successfully used, including 2 cases of male newborns without signs of feminization (158, 163-165).

Cabergoline has the most evidence of safety during pregnancy (based on its use in women with prolactinomas). However, it is less effective in reducing cortisol production, interferes with lactation, and is not applicable to cases of adrenal CS, the most common in pregnant women, or ECS. It has been effective in controlling hypercortisolism during pregnancy in 4 cases of CD (104, 165-167).

There is no experience with pasireotide, levoketoconazole, or osilodrostat during pregnancy, and they are currently contraindicated in their respective technical data sheets. Other drugs used for the treatment of CS are contraindicated in pregnancy. Mitotane is contraindicated because of its teratogenic potential and the risk of abortion (142). Additionally, due to its prolonged persistence in adipose tissue, it is recommended to postpone pregnancy even up to 5 years after completing treatment with this drug (168, 169). Mifepristone is contraindicated in pregnancy because of its antagonistic effect on progesterone receptors (it is actually indicated for the termination of unwanted or complicated pregnancies). This antiprogestogenic effect has not been observed in patients treated with relacorilant, but there is no experience of its use in pregnant women (8).

### Mild Autonomous Cortisol Secretion

In patients with an incidentally found adrenal adenoma MACS is defined by an after 1-mg overnight dexamethasone suppression test serum cortisol higher than 1.8 µg/dL without the specific clinical manifestations of overt CS, such as myopathy, bone fragility, and skin fragility (29). It is found in 20% to 50% of patients with adrenal incidentalomas (170). This condition is associated with an increased cardiometabolic risk and mortality when compared with nonfunctioning adrenal incidentalomas and the general population (171, 172).

The standard approach for patients with MACS is still controversial, but several studies demonstrate an improvement of the glucometabolic profile of patients with MACS after adrenalectomy (35, 173-175). In general, a surgical approach is recommended in patients with MACS who present associated comorbidities such as type 2 diabetes, hypertension, or osteoporosis, especially if the patient is young and the comorbidities are not adequately controlled when there is suppression of basal plasma ACTH (29). However, in those patients with surgical contraindications, with bilateral lesions, or who refuse surgery, steroidogenesis inhibitors may be considered when MACS-related comorbidities are not properly controlled (176).

In relation to the use of metyrapone in MACS patients, in a phase I/IIa, prospective study (Eudract no. 2012-002586-35) (177), 6 patients with MACS and 2 control groups of 6 sex-, age-, and body mass index-matched individuals were recruited to be treated with short-term metyrapone. Metyrapone led to a normalization of the disturbed circadian rhythm of cortisol when given in the late afternoon and evening (500 mg at 6 Pm and 250 mg at 10 Pm) in MACS patients. In addition, IL-6 levels were elevated before treatment (10 Pm to 2 Pm [area under the curve difference: 0.42 pg/mL/hour; *P* = .01]) and normalized posttreatment.

Ketoconazole, at a low dose (200-400 mg/day), also showed cortisol secretion normalization and BP improvement in a patient with bilateral adrenal macronodular hyperplasia and mild CS (178).

Mifepristone was evaluated in 3 different studies in patients with MACS, leading to an amelioration of insulin resistance, hypertension, quality of life, and cardiometabolic parameters with good tolerability (110, 179, 180). Debono et al (179) treated 6 patients with MACS with mifepristone 200 mg twice daily for 4 weeks and a significant reduction in insulin resistance was observed as 5 of 6 individuals showed a reduction in insulin, and in 2 patients, a clinically significant cardiovascular benefit was shown. In a second study, mifepristone was administered at doses of 300 mg daily over 6 months to 8 patients with MACS. This regimen led to a significant reduction in insulin resistance as measured and an improvement in Beck's Depression Inventory scores and Cushing's Quality of Life scores in most patients (180). In addition, another study included 4 patients with MACS and bilateral macronodular adrenal hyperplasia treated with mifepristone and an amelioration of glycemic control and hypertension as well as weight loss was observed in all patients (110).

Relacorilant was employed in 7 patients with ACTH-independent CS, including patients with MACS, and in the low-dose group (100-200 mg/day; n = 17), 5/12 patients (41.7%, 95% CI, 15.17-72.33) with hypertension and 2/13 patients (15.4%, 95% CI, 1.92-45.45) with hyperglycemia had a good response. However, the effects of relacorilant specifically on patients with MACS was not mentioned (125).

The number of evaluated patients remains, however, too low to draw any firm conclusion on the best medical treatment to consider for patients with MACS.

## Thromboprophylaxis in Endogenous Hypercortisolism and Management of Other Associated Comorbidities

### Thromboprophylaxis

One important consideration in patients with hypercortisolism is the high risk of venous thromboembolic events (VTE) in patients with active disease due to the well-documented association between CS and hypercoagulability (181, 182). Hypercortisolism is associated with increased production of procoagulant factors and activation of the coagulation cascade, as reflected by shortening of activated partial thromboplastin time, but also with impaired fibrinolytic capacity, indicated by an increased clot lysis time (183). In this regard, a systematic review of 48 studies and 7142 patients with CS reported an OR of spontaneous VTE in CS of 17.82 (95% CI, 15.24-20.85; *P* < .00001) when compared to a healthy population (182).

Regarding the impact of medical presurgical treatment on postsurgical outcomes, including the risk of thromboembolic events, the ERCUSYN trial did not observe differences in the prevalence of thromboembolism between the group of patients that received presurgical medical therapy and those not pretreated (22). The same group described that patients with CS had an elevated risk of developing thromboembolic events for a long period after surgery since 45% of the episodes occurred within 6 months since the operation, but episodes occurred until 10 years after the diagnosis of CS. Another important finding was that patients with thromboembolic events patients had higher UFC values than patients with CS without thromboembolic event diagnosis; thus, it may be expected that presurgical treatment could be associated with a reduction in the risk of these events. The ERCUSYN investigators propose that a VTE prevention protocol be widely adopted appears for patients with CS.

The increased VTE risk and the evidence of a reduction in the incidence of VTE among patients with CS who received thromboprophylaxis (184), led in 2025 to an international Delphi panel consensus and expert position statement with 14 recommendations. The most relevant are that (1) thromboprophylaxis should be considered at time of CS diagnosis in every patient and continued for 3 months after biochemical remission, provided there are no obvious contraindications; (2) The standard weight-based prophylactic dose of low-molecular-weight heparin should be the preferred agent for thromboprophylaxis in patients with CS; and (3) Perioperatively and around inferior petrosal sinus sampling, thromboprophylaxis should be reconsidered if not already initiated at diagnosis.

Although there is large practice variation regarding thromboprophylaxis management and perioperative medical treatment in patients with CS (185), the recent consensus statement on its use in patients with CS proposes that most cases should be treated, unless contraindications are present (186). Risk factors that increase the risk of VTE where thromboprophylaxis should be particularly considered include: active malignancy or cancer treatment, dehydration, sedentary lifestyle, smoking, use of oral estrogen replacement therapy, pregnancy or less than 6 weeks’ postpartum, age >60 years, known thrombophilia, obesity, 1 or more significant medical comorbidities, personal history or first-degree relatives with a history of VTE, or varicose veins with phlebitis.

#### Other comorbidities

Other frequent comorbidities associated with CS are cardiovascular and metabolic disorders, musculoskeletal alterations, as well as cognitive and mood impairment (187) (Table 3).

**Table 3.** Treatment of hypercortisolism related comorbidities

Comorbidity	Prevalence in CD	Specific considerations	Treatment
**Hypertension** (188)	50% to 95% (189-192)	Loss of the physiological nocturnal decrease is an early feature of HT in CS (193)	ACE-I or ARB as first-line treatment. Consider spironolactone or calcium channel blockers as an add-on treatment
**Diabetes mellitus** (194)	20% to 45% (189-191)	Glucose and insulin concentrations were higher in patients with CD compared with sex and age-matched controls and compared with BMI-matched controls after glucose loading (195)	Same recommendations than in type 2 diabetes. Dipeptidyl peptidase-4 inhibitor or a glucagon-like peptide-1 receptor agonist and insulin are especially useful for pasireotide-induced hyperglycemia (196)
**Obesity** (172)	25% to 100% (189-191)	Abdominal obesity, with preferential visceral rather than subcutaneous accumulation of fat tissue (197)	Diet recommendations and regular physical exercise within the possibilities of each patient. Consider glucagon-like peptide-1 receptor agonist or tirzepatide (especially when associated with diabetes mellitus) (198, 199)
**Dyslipidemia** (194)	16% to 60% (189-191)	Increase in total and LDL cholesterol and triglyceride concentrations, and reduced HDL cholesterol concentrations (195)	Similar treatment than in general population, but drug interactions with cortisol-lowering medications should be considered
**Osteoporosis and fractures** (200)	28% to 50% and 15% to 50% (201-204)	BMD is reduced in patients with CS (200)	Vitamin D and calcium supplementation. If medical treatment is indicated, oral bisphosphonates are the first line of treatment
**Thromboembolic risk** (205, 206)	15% to 18% (205, 207, 208)	Thromboprophylaxis should be used in the perioperative period in patients with high risk of VTE (5, 185)	Enoxaparin is most frequently used in active hypercortisolemia and postoperatively (184).
**Infections** (187, 209)	20% (210)	The impairment of immune function associated with active Cushing syndrome predisposes patients to infectious diseases, especially opportunistic infections. (210)	Vaccination against pneumococcus and influenza, strict hygiene measures, and, in some cases, antimicrobial prophylaxis are recommended

Abbreviations: ACE, angiotensin-converting enzyme; ARB, angiotensin receptor blocker; BMD, bone mineral density; CD, Cushing disease; CS, Cushing syndrome; VTE, thromboembolic events.

In general, the prevalence of these comorbidities and their severity are related to the degree of hypercortisolism (172). For example, comorbidities such as hypertension (up to 95%), hyperglycemia (20%-64%), and dyslipidemia (15%-60%) are well-known consequences of chronic cortisol excess and contribute to the increased cardiometabolic risk in CS (188, 194). The recommendations for the treatment of these comorbidities do not significantly differ from those provided for the general population.

Hypertension is a frequent associated comorbidity that occurs in 25% to 93% of patients with CS. It should be evaluated by 24-hour ambulatory monitoring. The main mechanisms involved in the pathogenesis of hypertension in CS are the modification induced by glucocorticoid excess in the renin-angiotensin system, the mineralocorticoid activity, the sympathetic nervous system, and the vasoregulatory system (187). Regarding HTN treatment, strict control of BP should be obtained (≤130/80 mm Hg) as stated in the guidelines of the European Society of Hypertension. The 11β-hydroxylase inhibitors, metyrapone and osilodrostat, can lead to a worsening of hypertension control by increasing cortisol and aldosterone precursors with mineralocorticoid activity (187). Overall, independent of the effect of the different cortisol-lowering medications, a specific pharmacological treatment for HTN is often required. Angiotensin-converting enzyme inhibitors or angiotensin receptor blockers are considered the first-line treatment. If proved ineffective, calcium channel blockers should be added. As an add-on treatment, spironolactone may be especially useful for patients with CS when there are associated hypokalemia (187, 188).

Diabetes mellitus is another common comorbidity in 10% to 50% of patients with CS (211, 212). As currently recommended for type 2 diabetes mellitus, the therapy should be patient-centered and not glycemia-centered. Glucose-lowering medications with a favorable cardiometabolic profile and associated with loss weight may be especially useful for patients with CS. In relation to medical treatment of hypercortisolism, it is important to remember that some drugs such as pasireotide causes frequent hyperglycemic events because the SSTR5 is expressed by pancreatic ß cells, which in turn control insulin production (49). On the other hand, cabergoline treatment is associated with improvement of diabetes mellitus and glucose intolerance by 60% and 46% (102). Similarly, ketoconazole, levoketoconazole, metyrapone, mifepristone, and osilodrostat have been associated with a positive impact on glucose metabolism (213).

Endogenous hypercortisolism has also a detrimental effect on bone health, with a prevalence of osteoporosis of 28% to 50% and of nontraumatic fractures of 15% to 50% (214). Most studies have shown that glucocorticoid bone loss and glucocorticoid-induced osteoporosis are potentially reversible after successful surgical treatment, although the time to bone recovery is relatively long and variable (215, 216). As a general recommendation, all patients with CS should be treated with adequate vitamin D and calcium supplementation. In addition to correcting concomitant risk factors for fractures, treatment with antiosteoporosis drugs may be necessary in active CS, with oral bisphosphonates being the preferred first-line treatment (217). Because of its high cost, teriparatide is usually reserved for the most severe forms of osteoporosis. As in the general population, anabolic agents are recommended as initial therapy for individuals at high or very high risk of fracture (218).

In cases of severe CS, there is an increased risk of opportunistic infections, including pneumonia, fungal infections, and sepsis, which can affect up to 25% of patients with CD, being 1 of the leading causes of mortality in these cases. Therefore, vaccination against pneumococcus and influenza, strict hygiene measures, and, in some cases, antimicrobial prophylaxis are recommended. Systematic prophylaxis against *Pneumocystis jirovecii*, a fungal pathogen causing pneumonia, with trimethoprim-sulfamethoxazole (800/160 mg) 3 times per week or 400/80 mg daily should be considered for patients with severe CS (3). In addition, the risk of severe COVID-19 infection in patients with CS appears to be higher than in the general population. For example, a recent cohort study found that the cumulative incidence of COVID-19 in patients with CD was 31.7%, compared to the general reference population (9.5%); the incidence was especially high in patients with active hypercortisolemia and obesity (219). Therefore, vaccination against SARS-CoV-2 should also be considered for patients with CS.

Other important health problems related to CS include neuropsychiatric disorders, such as impairment of cognitive function, depression, or mania; impairment of reproductive and sexual function; and dermatological manifestations (187).

#### Mortality

Patients with CS have an increased risk of overall and cardiovascular mortality, not only during the active phase of the disease but also after remission when compared to the general population (2, 210, 220-226). For example, in a series of 343 patients with CS and 34 000 controls, mortality risk was twice as high in patients with CS (hazard ratio [HR] 2.3; 95% CI, 1.8-2.9) (210). Similarly, a matched analysis of 371 patients with CD reported a higher overall mortality in patients than controls (HR 2.1; 95% CI, 1.5-2.8) with cardiovascular disease (48.5%) and infections (18.2%) being the most frequent causes of excess death (221). Another recent study of 609 cases with CS and 3018 matched controls detected that patients without remission within 2 years had a higher mortality risk than those achieving remission (HR 1.44; 95% CI, 1.00-2.17) (220). Another cohort study also found an increased standardized mortality ratio (SMR) in persistent cases at the last follow-up (SMR 4.99; 95% CI, 2.15-9.83; *P* < .001) whereas it was not higher in those in remission (SMR 1.66; 95% CI, 0.34-4.85; *P* = 0.543) (205, 227). A meta-analysis of 14 articles including 3691 patients (13 patients with CD and 7 with adrenal CS) described an overall SMR of 3.0 (95% CI, 2.3-3.9; *I*^2^ = 80.5%) for all etiologies of CS, 2.8 (95% CI, 2.1-3.7; *I*^2^ = 81.2%) for CD, and 3.3 (95% CI, 0.5-6.6; *I*^2^ = 77.9%) for adrenal CS (228). Mortality in patients with CS is also higher than in patients with other pituitary tumors. For example, 1 recent retrospective study comparing mortality in acromegaly, CD, macroprolactinomas, and nonfunctioning pituitary macroadenomas concluded that patients diagnosed with CD after 45 years of age had a significantly lower survival probability than other pituitary tumors subtypes in the first 15 years of follow-up (229). Germann et al also found that patients with CD showed increased hospitalization rates for psychiatric disorders (HR 3.27; 95% CI, 1.59-6.71) and a trend for sepsis (HR 3.15; 95% CI, .95-10.40) when compared to patients with nonfunctioning pituitary adenomas (230).

Mortality is higher in patients with ACC and ECS than in patients with benign causes of CS (187). However, in patients with ECS, survival varies considerably, mainly because of the underlying origin of the tumor, tumor stage, and severity of hypercortisolism. In this regard, a recent systematic review with 40 studies with a total of 1148 patients with ECS described that the 5-year survival probabilities for patients with pulmonary neuroendocrine neoplasm (NEN) was 81%, occult ECS 66%, thymic NEN 50%, and pancreatic NEN 40% (231). For patients with adrenal or persistent pituitary CS, a study of 83 patients with adrenal CS who underwent adrenalectomy (59 unilateral and 24 bilateral) found that postoperative mortality was increased in patients who underwent bilateral adrenalectomy (0% vs 8.3%; *P* = .081) (232).

Furthermore, an increase in the overall cancer risk has also been reported in patients with endogenous CS, mainly during the 10-year period following CS diagnosis. A recent cohort study with 609 patients with CS and 3018 age-, sex-, socioeconomic status-, and body mass index-matched controls found a cancer-related mortality at 10 years that was 2-fold higher in patients with CS, compared to controls, with major independent risk factors for cancer diagnosis within 10 years of initial confirmation of CS being age ≥60 years at CS diagnosis, male gender, and adrenal-origin of CS (233).

## Authors' Opinion For The Management of Hypercortisolism: Toward A Personalized Approach

As a summary, several points should be considered for the selection of the right drug for the right patient, in addition to the management of comorbidities (Table 4).

**Table 4.** Choosing the right drug for the right patient

	Yes	No
**Is the degree of hypercortisolism severe to life-threatening?**	Consider rapidly acting steroidogenesis inhibitors such as metyrapone (± ketoconazole) or osilodrostat for oral application, and etomidate for intravenous application in life-threatening hypercortisolism requiring intensive care.	Choose drug based on further questions.
**Is the patient pregnant?**	To date, no medical therapy for Cushing syndrome has been approved for pregnant or breastfeeding patients. However, metyrapone or cabergoline (in patients with Cushing disease) have been used in this scenario.	Choose drug based on further questions.
**Are there signs of clinical and/or biochemical hyperandrogenemia in female patients?**	Consider ketoconazole.	Choose drug based on further questions.
**Is there a preexisting (secondary) hypogonadism in male patients or a concern about inducing hypogonadism?**	Avoid ketoconazole.	Choose drug based on further questions.
**Is there a preexisting liver injury (Liver enzymes > 3×ULN) or a concern about hepatotoxicity?**	Avoid ketoconazole (234)	Choose drug based on further questions.
**In patients with mild Cushing disease, is there a concern about tumor size (macroadenoma, compressive symptoms)?**	Consider pasireotide or cabergoline.	Choose drug based on further questions.
**Is monotherapy likely to be ineffective or should the individual drug doses be reduced?**	Consider a combination, eg, metyrapone plus ketoconazole. In patients with Cushing disease, a combination of pasireotide plus cabergoline or ketoconazole plus cabergoline plus/or pasireotide may be considered (120)	Consider monotherapy.

### Which Drug For Which Indications? Some Insights on the Choice of the Drug and Dose

#### Severe hypercortisolism

Regardless of the cause, severe hypercortisolism should be treated with fast-acting cortisol-lowering drugs (235). Although studies have shown metyrapone to have a rapid onset of action, this has not been clearly demonstrated for osilodrostat. However, the authors' experience suggests that osilodrostat can also rapidly decrease cortisol concentrations if rapid uptitration is performed. In this setting, we suggest starting osilodrostat at 10 to 20 mg/day and rapidly uptitrating (increasing by 10 mg/day every 72 hours) based on morning cortisol concentrations (to evaluate AI in which cases glucocorticoid replacement should be initiated) and UFC concentrations (to evaluate efficacy). Dormoy et al (27) suggested that there is no theoretical maximum dose (up to 100 mg/day). Metyrapone should be initiated at 1500 mg/day (six 250-mg tablets), increasing by 3 tablets (ie, 750 mg) every 72 hours up to a maximum of 15 tablets/day (ie, 3750 mg/day), at the same rate as osilodrostat (236). Because of its shorter half-life, metyrapone should be administered 3 or 4 times per day (44). Combining metyrapone with ketoconazole (800 mg/day with a rapid increase up to 1200 mg/day) has been shown to be effective. This is especially useful when metyrapone alone does not rapidly decrease cortisol concentrations and in females with hyperandrogenism. Etomidate also acts very rapidly, but it should only be used for patients unable to take oral medications and in intensive care settings with continuous clinical (and biochemical) monitoring.

#### Moderate hypercortisolism

Moderate hypercortisolism is defined not only through levels of cortisol (UFC usually below 3- to 5-fold the ULN) but also from a clinical point of view (ie, lack of uncontrolled comorbidities). Moderate hypercortisolism does not usually require cortisol-lowering drugs to have a rapid effect. As such, a single treatment with the lowest recommended initial dose could be: 2 mg (or even 1 mg) twice daily for osilodrostat; 250 mg 3 times daily for metyrapone; 200 mg twice daily for ketoconazole; 10 mg once every 28 months for pasireotide; and 0.5 mg 3 times weekly for cabergoline. In patients well controlled by osilodrostat twice per day, switching to a lower, once-daily evening dose could be possible and help restore a more physiological cortisol rhythm if a close monitoring of cortisol secretion is maintained (78). Monitoring cortisol concentrations should be done every 2 to 3 weeks using UFC, morning serum cortisol, and LNSC (even, ideally, with salivary cortisol day profile), as detailed later. Clinicians should also keep in mind that osilodrostat has a second delayed efficacy happening about 3 weeks after initiation. This means that patients who are not well controlled a few days after the onset could be controlled with the same dose after 2 to 3 weeks. For osilodrostat specifically, waiting 3 weeks before increasing the dose is advised. Each treatment can be gradually increased until physiological cortisol concentrations are reached.

#### Specific indications of pituitary-targeted drugs

We advise that pituitary-targeted drugs such as pasireotide and cabergoline should be used in patients with CD for whom control of a tumor is necessary. This could be the case for patients with incomplete surgery and a large remnant. This is not a frequent situation, however. Clinicians should keep in mind that these treatments are not as effective as steroidogenesis inhibitors to control hypercortisolism, possibly requiring an off-label drug combination.

#### Gender-related choice of agent

Although an ACTH-targeted drug can be used equally in males and females, steroidogenesis inhibitors might be considered based on gender. Ketoconazole decreases androgens (237), whereas metyrapone and osilodrostat increase them; however, this effect seems less pronounced with osilodrostat (69). Therefore, ketoconazole may be preferred for females, whereas metyrapone or osilodrostat may be preferable for males. This effect is dose-dependent but can decrease over time, particularly with osilodrostat (71). This gender-related choice should not be taken into account in severe hypercortisolism, for which rapid control of cortisol secretion is the priority. Finally, as mifepristone is an androgen antagonist, it may cause hypogonadism in men.

### How to Monitor Medical Treatment?

The main aim of the treatment with a titration approach is to control cortisol secretion and avoid AI. This can be achieved through repeated measurements of UFC and morning blood cortisol levels. Once this is achieved, the treatment can be fine-tuned with repeated measurements of LNSC. However, this is not always accessible, and normal LNSC concentrations do not guarantee normal morning serum cortisol concentrations especially in patients with cortisol minimal diurnal variations. Salivary cortisol or cortisone day profiles improve assessing the diurnal cortisol rhythm and may be useful in chronotherapy, where higher drug doses are administered in the afternoon and/or evening. The optimal dose should strike a balance between mild over- or underdosing depending on the patient's profile and how they could handle adrenal crises.

In a block-and-replace approach, the main aim of the treatment is to induce AI so that it can be replaced with hydrocortisone. Once AI is achieved, we typically monitor serum cortisol concentrations at 8:00 Am, as this provides a good indication of daily cortisol production, assuming the treatment dosage is consistent throughout the day.

### The Risk of Adrenal Insufficiency

All treatments for hypercortisolism risk inducing AI. This can occur during the titration phase or during a period of stable dosing. At treatment initiation, all patients should be thoroughly informed about the clinical signs of AI, such as nausea, weight loss, intense fatigue, and low BP upon standing (i.e., postural hypotension). They should be educated on how to initiate glucocorticoid replacement therapy at home and receive an emergency pack containing the drug and materials for glucocorticoid injection. They should be educated together with relatives/friends on how to inject glucocorticoids. They should also know how to inform their endocrinologist (phone emergency numbers/email for specialist support) and when to go to the emergency department (same approach as for patients with other etiologies of AI). Patients should also receive written instructions about sick day rules (145).

During treatment, in cases of coexistent fever, infection, marked physical stress, or acute illness, sick day rules should apply (77). For patients who develop mild hypocortisolism, a medical evaluation should be performed to determine whether a temporary dose reduction of the steroidogenesis inhibitors for a few days may improve signs and symptoms. Additional glucocorticoid substitution should also be discussed. For cases of moderate-to-severe features of hypocortisolism, glucocorticoid replacement therapy should be rapidly initiated by the patient even before contact with the supervising medical team, and a temporary or definitive interruption of the cortisol-lowering drug be considered (58). It should be emphasized to patients and their carers that there is never a time when taking extra glucocorticoid for short periods (days) can do harm.

Furthermore, prolonged AI may occur after discontinuing osilodrostat; therefore, clinical data and morning cortisol concentrations must be monitored even after discontinuing the drug (Table 5). For example, a recent case reported a patient with ACTH-independent CS who developed medication-induced AI that has persisted for more than 3 years after only 4 months of treatment with osilodrostat (238). Another previous case reported a patient with CD who developed AI after 11 months of osilodrostat therapy; AI persisted for 23 months (239). In addition, 4 additional cases were previously described (Table 5) (240, 241). The mechanism leading to AI in patients treated with osilodrostat remains unclear, as this drug acts as a reversible inhibitor of 11β-hydroxylase with a half-life of only 4 hours. It could however be due to adrenal gland shrinkage (81).

**Table 5.** Cases of adrenal insufficiency following osilodrostat discontinuation

Author, year	Age and sex	Etiology of Cushing syndrome	Doses of osilodrostat	Duration of treatment	Duration of AI
Veloski C, 2025 (238)	38-year-old female	ACTH independent	6 mg/12 hours	4 months	36 months
Tejani S, 2024 (239)	41-year-old female	CD	3 mg/12 hours	11 months	23 months
Poirier J, 2023 (240)	Case 1: 51-year-old female	CD	2 mg/12 hours	6 months	1.4 months
	Case 2: 31-year-old female	CD	4 mg/12 hours	15 months	15 months
	Case 3: 41-year-old male	ACTH dependent CS	10 mg/12 hours	13 months	>10 months
Ferriere A, 2024 (241)	Case 1: 59-year-old sex NR	ACTH-dependent CS	20 mg/12 hours	14.5 months	12 months
	Case 2: 51-year-old female	CD	1 mg/12 hours	2.3 months	15 months

Abbreviations: AI, adrenal insufficiency; CD, Cushing disease; CS, Cushing syndrome; NR, not reported.

### Titration or Block and Replace

We report here a proposed modified algorithm to choose between the titration approach or block and replace:

- In patients with moderate hypercortisolism (mean of three UFC < 5×ULN), a slow titration approach with cortisol-lowering drugs and close monitoring should be preferred. The uptitration can be relatively slow to lower the risk of AI. In cases of poor clinical presentation with comorbidities and moderate hypercortisolism, a block and replace approach with faster uptitration should be considered.
- In patients with more intense hypercortisolism (UFC >5 but < 10 × ULN) but no life-threatening complications, the use of higher doses from the start and relatively fast-acting cortisol-lowering drugs (for instance, higher dose monotherapy with osilodrostat, metyrapone, or ketoconazole), with close monitoring of serum cortisol and/or 24-hour UFC concentrations every 5 to 7 days to adapt the dose is advised; an alternative is a block-and-replace approach.
- Finally, in cases with very intense hypercortisolism (UFC >10 × ULN) associated with serious complications that represent a medical emergency, it is recommended to use very high doses of fast-acting steroidogenesis inhibitors (preferably osilodrostat, or metyrapone, or a combination of ketoconazole and metyrapone) from the start in a block-and-replace approach, with rapid uptitration every 2 to 3 days, and in many cases as an in-patient in hospital.

## Conclusions

**Figure 6.**
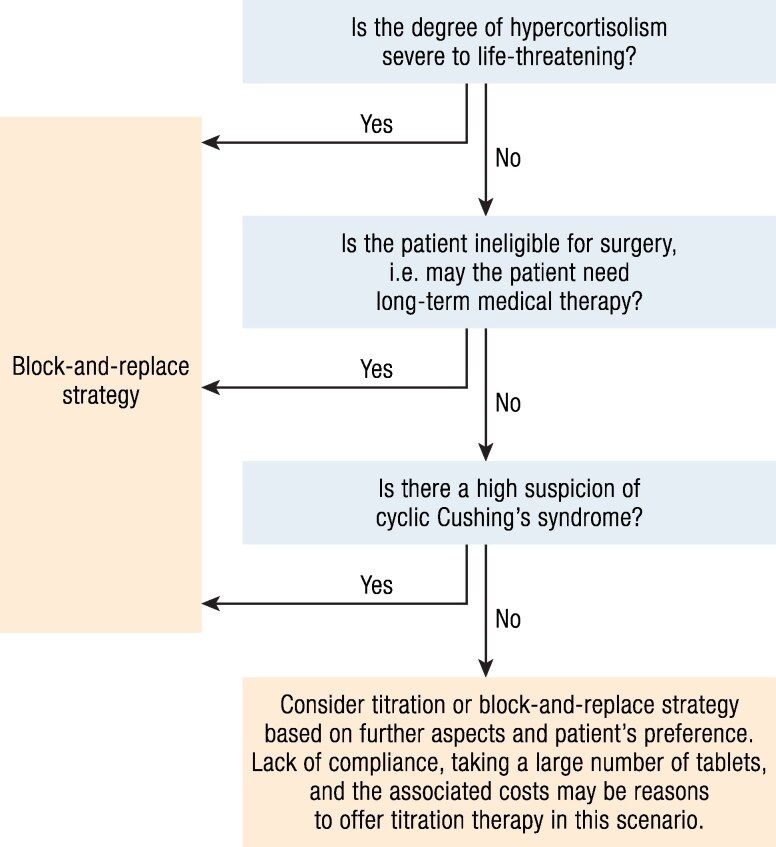
Choosing the right approach for the right patient: titration vs block-and-replace therapy.

**Figure 7.**
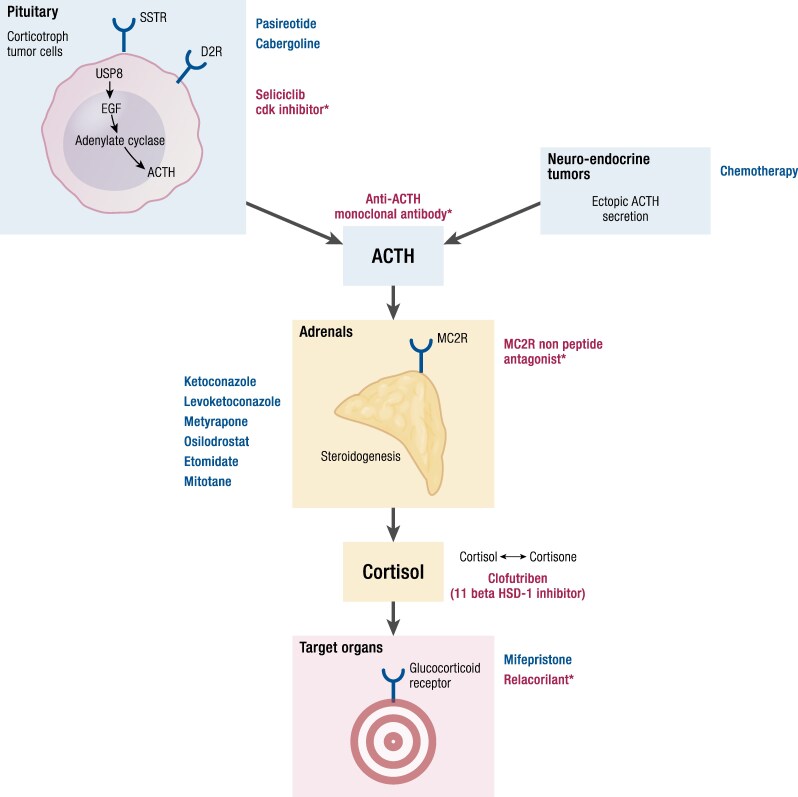
Summary figure—currently available drugs and potential new compounds for the treatment of Cushing syndrome. *Not available.

Medical treatment of hypercortisolism may be necessary in many situations in patients with CS. Currently there are 3 groups of medical therapies (adrenal steroidogenesis inhibitors, pituitary tumor-directed agents, and glucocorticoid receptor antagonists) with different therapeutic targets, efficacy, and safety profiles. In general, treatment with steroidogenesis inhibitors can be initiated in 1 of 2 ways: a titrating or a block-and-replace approach. An individualized approach based on the severity of hypercortisolism, comorbidities, and potential side effects as well as drug approval, availability, and costs should be considered to guide the selection of medical treatment for hypercortisolism control. There are some questions that may help determine both the therapeutic approach (titration vs block and replace) as well as the type of drug (Fig. 6 and Table 4) while waiting for possibly promising new compounds (Fig. 7).

## Abbreviations

ACC: adrenocortical carcinoma
AE: adverse event
AI: adrenal insufficiency
BP: blood pressure
cCH: cyclic Cushing syndrome
CD: Cushing disease
CRH: corticotropin-releasing hormone
CS: Cushing syndrome
DM: diabetes mellitus
DRD2: dopamine receptor subtype 2
ECS: ectopic Cushing syndrome
EGF: epidermal growth factor
EMA: European Medicines Agency
FDA: Food and Drug Administration
HR: hazard ratio
HTN: hypertension
IGT: impaired glucose tolerance
LAR: long acting release
LNSC: late night salivary cortisol
MACS: mild autonomous cortisol secretion
NEN: neuroendocrine neoplasm
SMR: standardized mortality ratio
SSTR: somatostatin receptor
UFC: urinary free cortisol
ULN: upper limit of normal
